# Transcriptome Profiling Revealed Stress-Induced and Disease Resistance Genes Up-Regulated in PRSV Resistant Transgenic Papaya

**DOI:** 10.3389/fpls.2016.00855

**Published:** 2016-06-16

**Authors:** Jingping Fang, Aiting Lin, Weijing Qiu, Hanyang Cai, Muhammad Umar, Rukai Chen, Ray Ming

**Affiliations:** ^1^Key Lab of Sugarcane Biology and Genetic Breeding, Ministry of Agriculture, Fujian Agriculture and Forestry UniversityFuzhou, China; ^2^FAFU and UIUC-SIB Joint Center for Genomics and Biotechnology, Fujian Agriculture and Forestry UniversityFuzhou, China; ^3^Department of Plant Biology, University of Illinois at Urbana-ChampaignUrbana, IL, USA

**Keywords:** *Carica papaya* L., *papaya ringspot virus* (PRSV), transgenic papaya, differentially expressed gene, alternative splicing

## Abstract

Papaya is a productive and nutritious tropical fruit. *Papaya Ringspot Virus* (PRSV) is the most devastating pathogen threatening papaya production worldwide. Development of transgenic resistant varieties is the most effective strategy to control this disease. However, little is known about the genome-wide functional changes induced by particle bombardment transformation. We conducted transcriptome sequencing of PRSV resistant transgenic papaya SunUp and its PRSV susceptible progenitor Sunset to compare the transcriptional changes in young healthy leaves prior to infection with PRSV. In total, 20,700 transcripts were identified, and 842 differentially expressed genes (DEGs) randomly distributed among papaya chromosomes. Gene ontology (GO) category analysis revealed that microtubule-related categories were highly enriched among these DEGs. Numerous DEGs related to various transcription factors, transporters and hormone biosynthesis showed clear differences between the two cultivars, and most were up-regulated in transgenic papaya. Many known and novel stress-induced and disease-resistance genes were most highly expressed in SunUp, including *MYB, WRKY, ERF, NAC*, nitrate and zinc transporters, and genes involved in the abscisic acid, salicylic acid, and ethylene signaling pathways. We also identified 67,686 alternative splicing (AS) events in Sunset and 68,455 AS events in SunUp, mapping to 10,994 and 10,995 papaya annotated genes, respectively. GO enrichment for the genes displaying AS events exclusively in Sunset was significantly different from those in SunUp. Transcriptomes in Sunset and transgenic SunUp are very similar with noteworthy differences, which increased PRSV-resistance in transgenic papaya. No detrimental pathways and allergenic or toxic proteins were induced on a genome-wide scale in transgenic SunUp. Our results provide a foundation for unraveling the mechanism of PRSV resistance in transgenic papaya.

## Introduction

Papaya (*Carica papaya* L.) is a major tropical fruit crop with outstanding nutritional and medicinal values. Various aspects of genomics and genetics are relatively easy with papaya owing to its small genome (2*n* = 18, 372 Mb), short generation time, incipient sex chromosome, and established transformation system (Arumuganathan and Earle, 1991; Ming et al., 2008; Wang et al., 2012). The lack of a recent genome wide duplication in papaya helps to study angiosperm genome evolution (Ming et al., 2012). In addition, papaya is in the order Brassicales with the model plant *Arabidopsis thaliana* that diverged about 72 million years ago, facilitating comparative structural and evolutional genomic research in both species (Wikström et al., 2001). These characteristics make papaya an excellent model for tree fruit species.

*Papaya Ringspot Virus* (PRSV) is the most devastating threat to year-round production of papaya in the world. PRSV is a single-stranded positive sense RNA virus that belongs to the genus *Potyvirus* of family Potyviridae (Tripathi et al., 2008). It can be easily transmitted in a non-persistent manner by aphids and mechanical means, but difficult to control due to the lack of naturally resistant germplasm from *Carica papaya* (Gonsalves, 1998; Ming and Moore, 2014). The infected papaya plants are characterized by abnormally stunted growth, malformed yellowing leaves with mosaic and reduction of fruit. Other symptoms such as water soaked oily streaks on petioles, dark green streaks on trunks, round spots and bumps on fruit are also indicative of this devastating viral disease. The papaya industry in Hawaii has been severely damaged since the onset of PRSV's spread in 1992. Hawaii's production of marketable papaya drastically declined from 55.8 million pounds in 1992 to 33.6 million pounds in 1998. None of the approaches aiming at creating papaya resistant germplasm by crossing it to wild relatives (Manshardt and Wenslaff, 1989) or cross-protection (Yeh and Gonsalves, 1984; Mau et al., 1989) were able to allow the papaya industry to recover. In 1998, the deregulation and commercialization of PRSV-resistant transgenic papaya, SunUp and Rainbow, revived the industry (Tripathi et al., 2008). Today, transgenic papaya acreage is about 85% of the total in the state of Hawaii.

Genetically engineered (GE) papaya plants were developed based on the concept of pathogen-derived resistance (PDR) using PRSV coat protein (*cp*) gene transformation to protect the host plants against PRSV infection (Sanford and Johnston, 1985; Fitch et al., 1992). Post transcriptional gene silencing (PTGS) or RNA interference (RNAi) is a sequence-specific mRNA degradation mechanism by which plants are able to protect themselves from viral infection by targeting viral transgenes (Vaucheret et al., 1998, 2001). The PRSV-resistant papaya SunUp is essentially a genetically engineered version of the existing Sunset cultivar. Sunset is a pink-fleshed cultivar with a low level of residual heterozygosity due to over 25 generations of inbreeding (Storey et al., 1969). SunUp is derived from the transgenic line 55-1 R_0_ which was obtained via microprojectile bombardment-mediated transformation of 2,4-D-treated immature zygotic embryos with a plasmid construction containing the neophosphotransferase II (*nptII*) and β-glucuronidase (*uidA*; *gus*) genes flanking a PRV HA 5-1 *cp* gene expression cassette (Fitch et al., 1990, 1992). SunUp is a cultivar homozygous for the PRSV CP transgene and is highly inbred with a heterozygosity of only 0.06% (Ming et al., 2008). Rainbow is an F1 hybrid hemizygous for the PRSV CP transgene resulting from a cross between SunUp and the yellow-fleshed non-GE cultivar Kapoho (Manshardt, 1999). SunUp and Sunset have separated for more than 20 years, i.e., more than 20 meiosis, and they share lots of phenotypic and functional features. SunUp plants grow phenotypically normal and both cultivars have the same fruit appearance of pink-tinged flesh with a melting texture (Figure S1C). The total soluble solids of SunUp fruit were within the expected range (Ming and Moore, 2014). It is obviously that transgenic papaya showed excellent resistance to PRSV (Figure S1B). However, we also found in our research that even at the initial stage of growth prior to infection with PRSV, seedlings of transgenic papaya SunUp exhibited better growth performance compared with that of its progenitor Sunset under the same conditions (Figure S1A). Their increased growth rate was particularly pronounced from ~3 weeks after germination.

Although transgenic papaya reversed the downward trend of papaya production in Hawaii, papaya production has not yet returned to its 1992 levels. Perhaps the primary reason is that this industry still faces some challenges in bringing transgenic papaya to markets outside the US. Taking biosafety into account, some countries have zero tolerance policies for transgenic papaya. Overall, potential allergenicity of this new product and possible altered nutritional composition are the two main areas of concern in regard to the food safety of GE organisms. There is no publicly available evidence to date that the coat protein of PRSV or other plant viruses is allergenic or detrimental to human health in any way. Detailed studies on the potential of the *cp* gene as an allergen showed that the transgene-derived PRSV CP does not pose a risk of food allergy following accepted allergenicity assessment criteria (Fermín et al., 2011). In the toxicology assessment, CP was not considered a novel protein due to the history of human consumption of PRSV-infected plants without adverse health effects. Measured amounts of CP in transgenic plants were even much lower than those of infected plants. No differences were observed between GE and non-GE papaya for 36 nutrients at any of the tested fruit ripeness stages (Tripathi et al., 2011). Transgene number or vector elements inserts were characterized as part of a petition to Japan to allow import of these transgenic papaya fruit from Hawaii. Three stably inherited transgene inserts were detected in Rainbow and SunUp by Southern blot analysis (Suzuki et al., 2008). One was a 9789 bp functional insert, coding for the PRSV *cp, nptII*, and *uidA*; two were unintended inserts: a partial *nptII* gene fragment and a partial *tetA* gene fragment. Intriguingly, five out of six transgene flanking sequences were chloroplast-derived, and one was mitochondria-derived. Four of the flanking sequences were closely associated with topoisomerase I recognition sites. From analysis of the insertion site and flanking genomic DNA, no changes to endogenous gene function and no allergenic or toxic proteins were predicted. Polymerase Chain Reaction (PCR) and Southern blot analysis are the most commonly used methods to detect integration of vector sequences at the genome structure level. However, at the genome function level, how genome-wide gene expression is altered and whether interrupted genes are induced by bombardment remained to be determined. Proving no detrimental functional changes induced at the genome-wide level in GE papaya is an essential criterion in the context of genetically modified organism (GMO) biosafety regulation. Moreover, DNA introduction into a host organism via different methods such as agrobacterium-mediated and particle bombardment transformation can cause varied single nucleotide polymorphism (SNPs), insertions/deletions (InDels), rearrangement and integration positions, affecting both transgene expression and the potential for gene disruption in different ways (Kohli et al., 2003; Latham et al., 2006; Wilson et al., 2006). Therefore, a better understanding of how bombardment affects plant genome will further our understanding to develop more reliable methods for crop improvement.

The last decade has seen revolutionary advances in DNA and RNA sequencing technologies with the advent of Next Generation Sequencing (NGS) techniques (Metzker, 2010). RNA-sequencing (RNA-Seq) is an attractive analytical tool in transcriptomics. It is well established and developed very rapidly, decreasing the running costs and playing a central role for unraveling the complexity of gene expression regulation (Morozova and Marra, 2008). A female SunUp genome was sequenced and assembled using whole-genome shotgun (WGS) sequencing, small-insert libraries and Sanger sequencing approaches with integration of physical and genetic maps in 2008 (Ming et al., 2008). This is the first transgenic crop to be sequenced. This genome spans 372 Mb including embedded gaps, representing 75% of papaya genome, 90% of the euchromatic regions and 92% of the papaya ESTs. The availability of the SunUp draft genome and the development of RNA-Seq technology have enabled us in this study to visualize the landscape of changes occurring in transcriptome of SunUp after transformation of its progenitor Sunset with the aim of unraveling the global impact of particle bombardment-mediated transformation on whole genome function. The comparison of dynamic transcriptome expression profiles between these two cultivars may shed light on the potential gene disruption and changes of expression caused by genome structure variation after transformation and give evidence at the transcriptional level that genetically modified papaya are not harmful concerning the biosafety of GMOs.

## Materials and methods

### Plant materials and growth conditions

Seeds of transgenic papaya cultivar SunUp and donor control Sunset were planted and grown in plastic pots filled with organic loam in a greenhouse in March, 2015. Greenhouse temperature was set at 27°C. Sixty pots (one seedling per pot, 30 pots per cultivar) were used in this experiment. Plants were watered every day. After 68 days, when the plants had 5–7 leaves, the plantlets were transplanted to field at Fujian Agriculture and Forestry University (FAFU), Fuzhou, China. Young and healthy leaf tissue from 4-month-old plants was collected for RNA extraction. All plants tested PRSV-negative. Three biological replicates were analyzed for each cultivar.

### RNA extraction and library construction

Total RNA was extracted from ground leaf using RNAprep pure Plant Kit (Tiangen, #DP432), according to the manufacturer's protocol. Residual genomic DNA was removed with RNase-Free DNase I (Code No. 2212, Takara). The quality of total RNAs was verified on an Agilent 2100 Bioanalyzer (Plant RNA Nano Chip, Agilent). After quantity and quality determination, a single indexed RNA-Seq library was constructed using NEBNext Ultra™ RNA Library Prep Kit for illumina (NEB, #E7530), and then sequenced by Illumina HiSeq2500 in paired-end 150 nt mode. Prior to downstream processing, raw reads generated by Illumina HiSeq2500 were initially processed to obtain clean reads by removing adaptor sequences, empty reads, and low quality sequences (>50% of the bases with a quality score ≤ 5).

### Sequencing read mapping and gene expression estimation

Sequences of three SunUp transformation plasmid derived inserts with genomic borders are available at Genbank (accession numbers: FJ467933, FJ467932, and FJ467934). We then concatenate these three sequences with the papaya “SunUp” reference genome (http://www.plantgdb.org/CpGDB/) which does not include the sequences of transgene inserts. The trimmed paired-end reads of each sample were aligned to the new assembled papaya “SunUp” reference genome using TopHat v2.0.14 default settings (Trapnell et al., 2012). The reads for each biological replicate were mapped independently, and reads that mapped to reference sequences from multiple genes were filtered out. The resulting Binary Alignment/Map (BAM) alignment files were provided to Cufflinks v2.2.1 to generate a transcriptome assembly for each replicate. These transcriptome assembly files were then merged with the reference transcriptome annotation into one unified annotation by using the Cufflinks *cuffmerge* utility for further annotation and differential expression analysis. The RNA-Seq read-mapping result was used to predict gene expression profiles, and the FPKM (Fragments per kilobase of exon per million fragments mapped) value of each sample were estimated by Cufflinks. To avoid false positive estimation of gene expression, transcripts with FPKM values < 1 in both libraries were not subjected to further analysis.

### Identification of differentially expressed genes

Differentially expressed gene and isoform analysis of the mapping results was analyzed and calculated using the Cufflinks *cuffdiff* program. *Cuffdiff* is an independent, widely used tool which takes a nonparametric, annotation-guided approach to estimate the means and variances of transcript FPKM values under different conditions, using Student's *t*-tests to identify differentially expressed transcripts. *Cuffdiff* allows taking multiple technical or biological replicate sequencing libraries per condition (Trapnell et al., 2012). The BAM alignment files and the merged annotation are fed to *cuffdiff* to calculate expression levels in two samples and test the statistical significance of observed changes in expression between them. An absolute log_2_ (FPKM_SU−NP_/FPKM_SS−NP_) >2 (fold change >2) and an adjusted *p*-value (FDR, false discovery rate) < 0.05 were used as thresholds to identify significant differences in gene expression. CummeRbund (http://compbio.mit.edu/cummeRbund/), an extension R package of the cufflinks, was used for visualization of results and read dispersion.

### Functional annotation

Sequence-similarity Blast searches of all papaya predicted protein sequences were conducted with a typical cut-off *E*-value of 10^−5^ against several publicly available protein databases: the National Center for Biotechnology Information (NCBI) non-redundant (Nr) protein database, Clusters of Orthologous Groups (COGs), and Kyoto Encyclopedia of Genes and Genomes (KEGG). Gene Ontology (GO) terms describing biological processes, molecular functions and cellular components were assigned to the predicted genes by Blast2GO program (Conesa et al., 2005) based on the Nr blastp output. Functional classification of GO and COG for all genes was performed by in-house Perl scripts.

For the comparative analysis of DEGs between PRSV susceptible and resistant transgenic papaya cultivars, all DEGs identified in this pairwise comparison were used for GO and KO enrichment analysis. Single enrichment analysis (SEA) was performed in agriGO program (Du et al., 2010) using the gene models of papaya reference genome as background. The Fisher statistical test was applied to test for enrichment of functional categories with Bonferroni's correction (FDR ≤ 0.05). KEGG metabolic pathway annotation and enrichment of the DEGs were performed by using KOBAS (KEGG Orthology Based Annotation System) (Xie et al., 2011). The hypergeometric test was applied with Benjamini and Hochberg's correction method at an FDR of 5%.

Differential expression genes were classified as genes encoding transcription factors (TFs), transporter proteins (TPs) and hormone-related genes according to PlantTFDB v3.0 (Jin et al., 2014), TransportDB (Ren et al., 2007), and the Arabidopsis Hormone Database 2.0 (Jiang et al., 2011). The log_2_-transformed (FPKM) values for each transcription factor, transporter, and hormone-related gene were used to generate heat maps in R version 3.2.1 statistical package (www.CRAN.R-project.org).

### Validation of differentially expressed genes by qRT-PCR

A total of 21 candidate DEGs between Sunset and SunUp were selected for quantitative real-time PCR (qRT-PCR) assays (Table S7). Approximately 1 μg of total RNA per sample was treated with DNase I (Takara, Dalian, China), and then reverse-transcribed using a Prime-Script 1st Strand cDNA Synthesis Kit (Takara, Dalian, China) following the manufacturer's protocol. A total of 1 μl of 10-fold diluted cDNAs were used as template in 20 μl PCR reaction mixture containing 10 μl of 2 × SYBR® Green I Master Mix (Takara, Dalian, China), 0.4 μM of each primer. Gene specific qRT-PCR Primers (See Table S8) were designed from coding sequences of all candidate genes using IDT SciTools® Web Tools (Owczarzy et al., 2008). The qRT-PCR reactions were performed on a CFX Connect Real-Time System (Bio-Rad, Singapore) under the following conditions: one cycle at 95°C for 30 s, followed by 42 cycles at 95°C for 5 s and 60°C for 30 s. Melting curve analysis was added at the end of 42 cycles to check the amplification specificity of target fragments. Three biological replicates were performed for each gene in order to exclude sampling errors. The reference housekeeping gene *EIF* (Eukaryotic initiation factor 4A) (Zhu et al., 2012) was used as the internal control. Relative expression levels of the selected DEGs were calculated using the 2^−ΔΔCt^ method (Schmittgen and Livak, 2008), and *EIF* was used to normalize the amount of template cDNA in each reaction.

### Alternative splicing identification and classification

To identify alternative mRNA processing events and their corresponding types in each cultivar, in-house Perl scripts (available upon request) were used to predict AS events and determine the types of the events from the exon-exon splice junctions' bed files output by TopHat. Three biological replicates were combined and aligned against the reference genome in order to find all possible splice junctions between annotated exons for each gene in a cultivar. Six different alternative splicing types were identified by comparing RNA-Seq reads with annotated gene models, namely skipped exon (SE), retained intron (RI), alternative 5′ splice site (A5SS), alternative 3′ splice site (A3SS), alternative 5′ UTR splice (5UTR), and alternative 3′ UTR splice (3UTR). They were extracted for further analyses.

## Results

### Analysis and mapping of illumina sequencing reads

**Figure 1:**
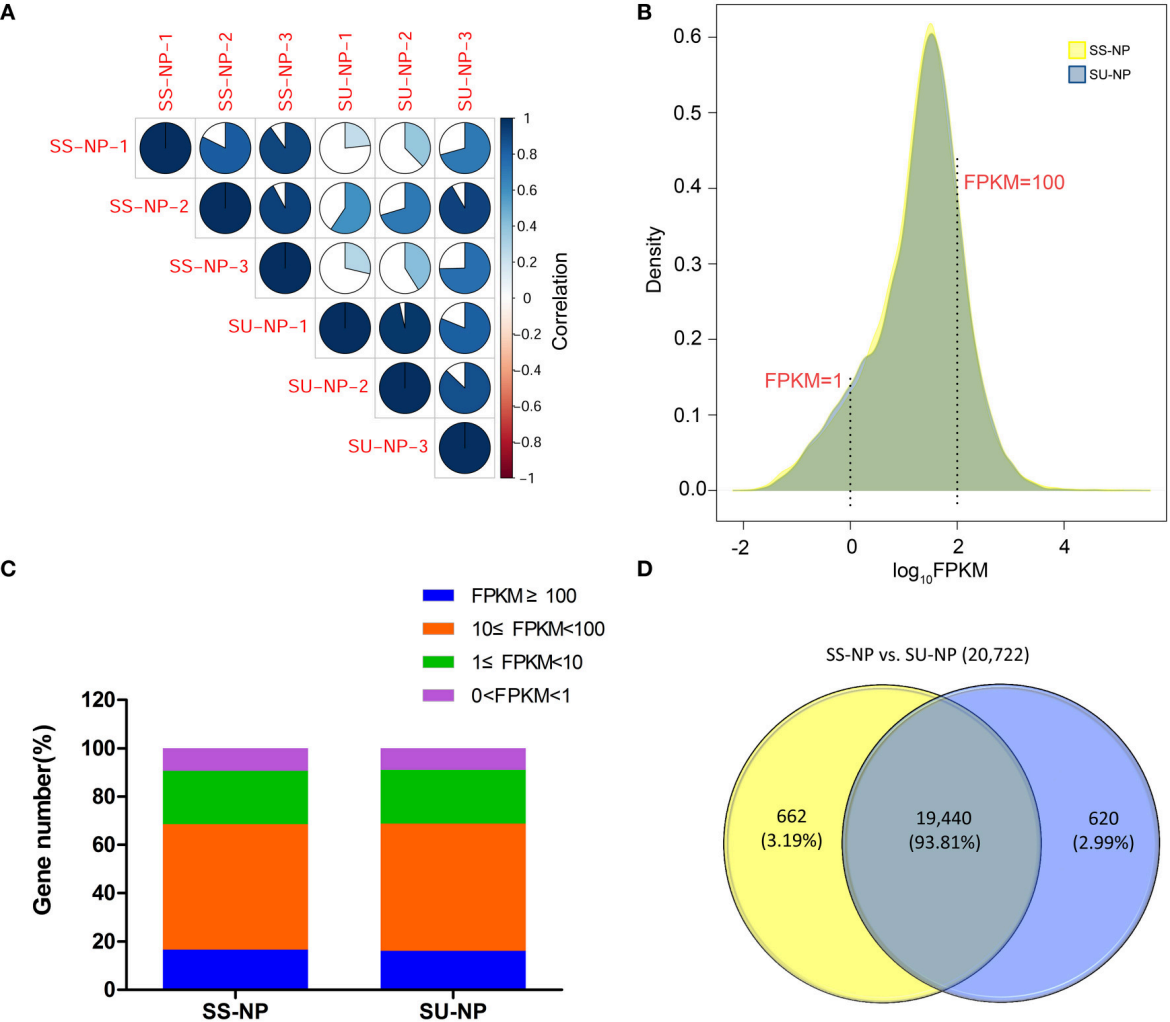
**Overview of papaya PRSV-negative SunUp and Sunset transcriptomes. (A)** Pairwise correlation of different biological replicates from SS-NP and SU-NP using FPKM values. The color intensities (scale in the side bar) and the numbers indicate the degree of pairwise correlation. **(B)** log_10_FPKM distributions of genes for SS-NP and SU-NP. Yellow and purple represent Sunset and SunUp, respectively. **(C)** Percentage of genes expressed in each variety. **(D)** Venn diagrams showing mapped genes expressed in SS-NP and SU-NP.

Sunset is a pink-tinged flesh cultivar that is susceptible to PRSV, and SunUp is a PRSV-resistant papaya cultivar that is essentially a transgenic Sunset. SunUp plants showed vigorous growth compared to Sunset plants (Figure S1A). No abnormal phenotypes were observed in SunUp. To characterize transcriptomic changes at the genome scale induced by microprojectile bombardment, we used the Illumina2500 sequencing platform to sequence SunUp and Sunset transcriptomes, including three PRSV-negative Sunset leaf samples (Sunset non-PRSV leaves, SS-NP) and three PRSV-negative SunUp leaf samples (SunUp non-PRSV leaves, SU-NP). Sequencing generated 342.7 million reads, a total raw dataset of 51.8 Gb. After removing low-quality sequences and trimming adapter sequences, over 342.1 million paired-end clean reads were retained from all libraries, with the number of RNA-Seq reads per library ranging from 45.9 to 64.1 million (Table 1). Mapping to the available papaya SunUp genome, transcript assembly, and quantification were performed by using TopHat and Cufflinks. After mapping the sequence reads against the reference genome, approximately 80% of the total reads matched unique genomic locations (74.9–76%), while the remainders had either multiple matches (3.2–4.5%) or remained unaligned (20.5–20.8%). Only uniquely mapped reads were used in the analysis of gene expression in different cultivars. Before downstream analysis, the correlation coefficient between each pair of three biological replicates was evaluated by comparing their RNA-Seq expression profiles (Figure 1A). This shows that the estimated levels of gene expression were highly consistent between any replicate pair of each cultivar (*r* = 0.81 − 0.96). In total, 20,700 transcripts were identified from both conditions, accounting for 74.1% of the annotated genes in papaya. In both Sunset and SunUp transcriptomes, the number of mapped genes in Sunset was found to be similar to that in SunUp (20,102 vs. 20,060) (Table 1). Among these genes, more than 74% had FPKM values in the range of 1–100 for each cultivar (Figures 1B,C), and it was observed that the FPKM ranges in two cultivars were almost similar. The log_10_FPKM shows a primary peak of high expression genes, with two shoulders of low-expression transcripts (Figure 1B). We further compared the mapped genes between Sunset and SunUp transcriptomes, and found that 93.81% of mapped genes were common in two cultivars, but up to 662 and 620 genes were specifically expressed in Sunset and SunUp, respectively (Figure 1D).

**Table 1:** **Summary of the Illumina 2500 sequencing reads and their matches in the papaya genome**.

**Samples**	**Biological replicate**	**Total raw reads**	**Total clean reads**	**Unmapped reads (%)**	**Mapped reads**		**Matched genes (%)**
					**Unique (%)**	**Non-unique (%)**	
SS-NP	1	62,647,390	62,531,002	12,952,411 (20.7)	47,429,221 (75.8)	2,149,370 (3.4)	20102 (71.9)
	2	64,194,178	64,073,984	13,117,339 (20.5)	48,212,482 (75.2)	2,744,163 (4.3)	
	3	53,744,902	53,651,186	11,054,605 (20.6)	40,178,028 (74.9)	2,418,553 (4.5)	
SU-NP	1	46,021,706	45,937,939	9,557,946 (20.8)	34,841,827 (75.8)	1,538,166 (3.3)	20060 (71.8)
	2	59,345,134	59,240,353	12,283,931 (20.7)	45,040,771 (76.0)	1,915,651 (3.2)	
	3	56,746,644	56,648,778	11,799,675 (20.8)	42,720,172 (75.4)	2,128,931 (3.8)	

*RNA-Seq reads mapped to papaya reference genome by using TopHat*.

### Annotation, functional classification, and KEGG analysis of DEGs

**Figure 2:**
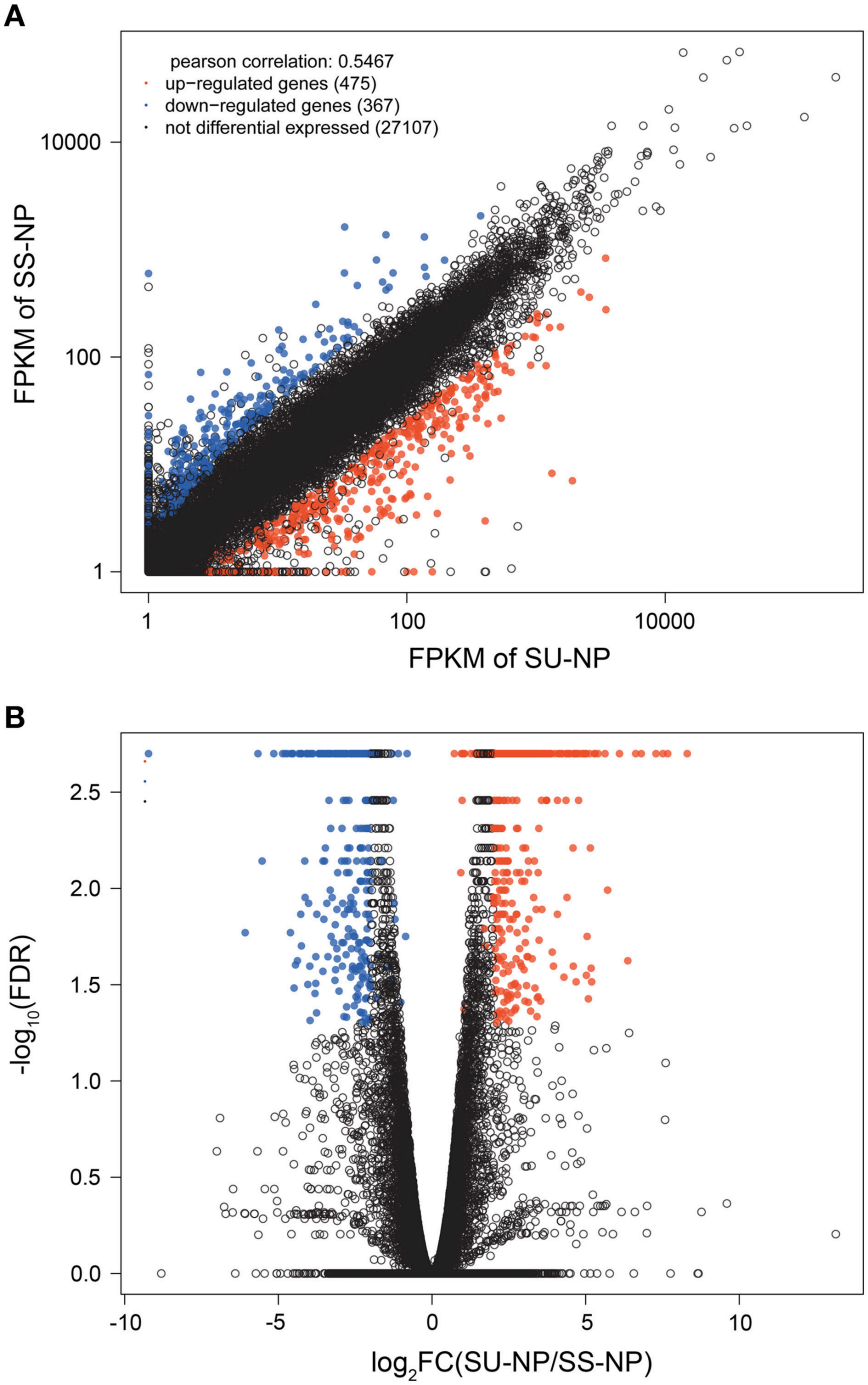
**Visualization of expression changes in different libraries. (A)** Scatter plot and **(B)** Volcano plot of the transcriptomes of PRSV-negative Sunset and SunUp. In the scatter plot, FPKM values in donor control (*Y*-axis) have been plotted against FPKM values of transgenic papaya (*X*-axis). In the volcano plot, statistical significance (−log_10_ of adjusted *p*-value; *Y*-axis) have been plotted against log_2_ fold change (*X*-axis). Significantly up-regulated genes are represented by red dots, while down-regulated genes are represented by blue dots.

To identify global transcriptional changes occurring after bombardment of the transgene, we applied *cuffdiff* to identify genes that were differentially expressed between PRSV-negative SunUp and its progenitor cultivar Sunset. Expression profiles of differentially expressed genes (DEGs) are expected to meet the following three criteria: (i) the FPKM value is ≥1 in either of the libraries, (ii) log_2_ (FPKM_SU−NP_/FPKM_SS−NP_) is >2 or < −2, and (iii) the adjusted *p*-value is < 0.05. In this study, DEGs with higher expression levels in SunUp than in Sunset were defined “up-regulated genes,” whereas those with lower expression levels in SunUp were referred to as “down-regulated genes.” As shown in the scatter plot (Figure 2A), transcript abundance in both Sunset and SunUp are similar for most of the transcripts, which is consistent with Figures 1B,C. To determine how many genes are significantly regulated, a volcano plot was constructed by plotting the fold change values against the negative log_10_ of adjusted *p*-values (Figure 2B). The higher the negative log-transformed FDR, the more significant the regulation. A fold change of zero is in the middle of the volcano. On the left side, negative fold change values indicate down-regulation, while on the other side are the positive fold change values thereby indicating up-regulation. In total, 842 DEGs were identified showing up- or down- regulation between Sunset and SunUp, in which 475 genes were up-regulated, and 367 were down-regulated. The DEGs were found to be randomly distributed amongst the 9 papaya chromosomes (Table 2). Chromosome 3 and 8 displayed a relatively higher rate of DEGs than others, while chromosome 2 and 9 contained the least.

**Table 2:** **The distribution of differentially expressed genes along papaya chromosomes**.

**Chrom**.	**Total size (bp)**	**No. of genes**	**No. of DEGs**	**Percent of DEGs (%)**
CHROM_1	22,976,894	2088	78	3.74
CHROM_2	28,675,255	2424	66	2.72
CHROM_3	29,397,938	2526	102	4.04
CHROM_4	27,056,416	2417	91	3.76
CHROM_5	24,352,217	1794	58	3.23
CHROM_6	30,516,430	2339	71	3.04
CHROM_7	22,375,162	1740	54	3.10
CHROM_8	21,952,264	1829	72	3.94
CHROM_9	27,303,179	1969	55	2.79
Unanchored scaffolds	135,176,073	8829	195	2.21
SunUp reference genome	369,781,828	27,955	842	3.01

**Figure 3:**
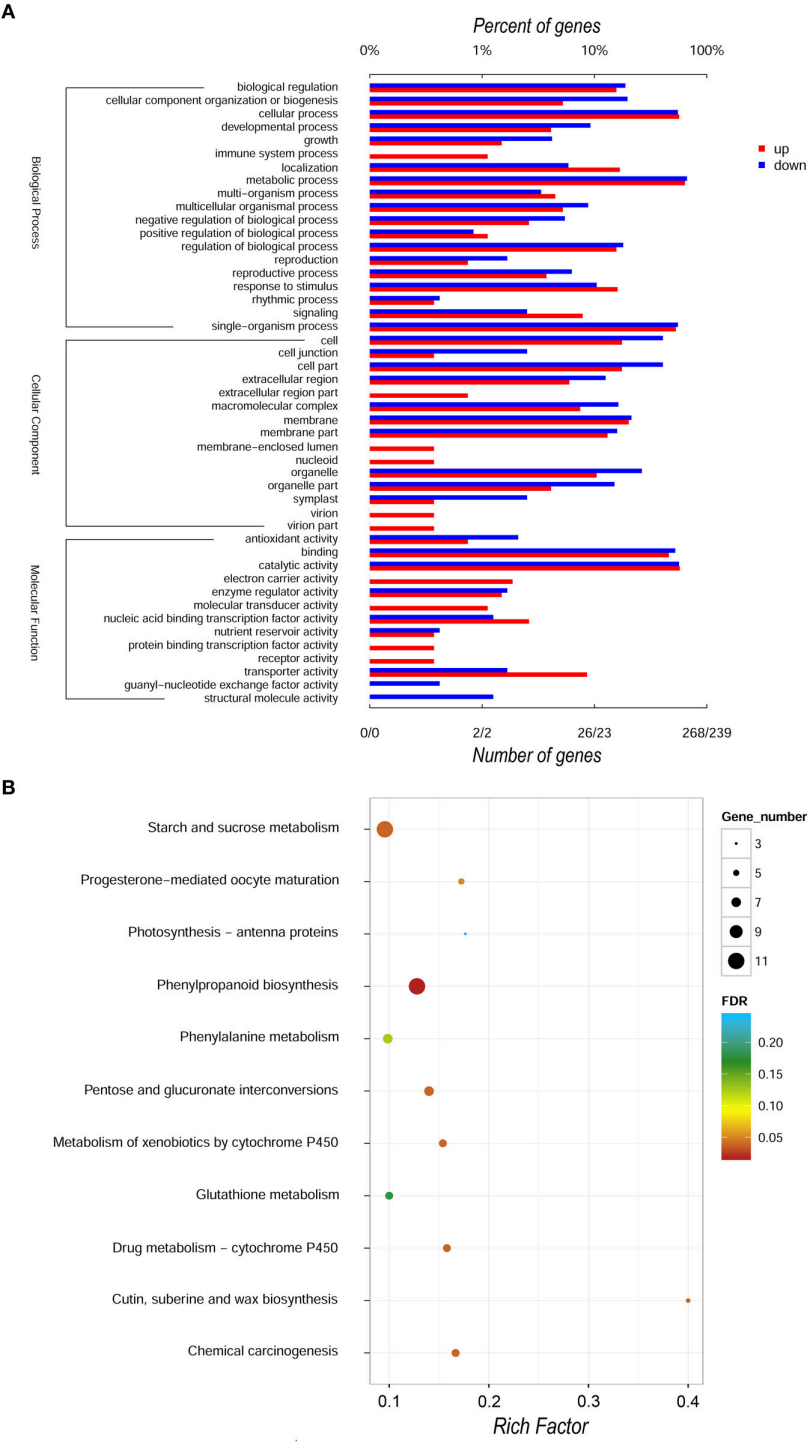
**GO and KEGG annotation of DEGs comparison of SunUp and Sunset transcriptomes. (A)** Level 2 GO annotation of up-regulated and down-regulated genes. We divided the sets into three major GO ontologies: biological process, cellular component and molecular function. Red and blue bars represent the number and percentage of up-regulated and down-regulated genes, respectively. **(B)** A KEGG pathway enrichment scatter diagram of DEGs. Only the top 11 most strongly represented pathways were displayed in the diagram. The degree of KEGG pathway enrichment was represented by a rich factor, the FDR, and the number of DEGs enriched in a KEGG pathway. The rich factor indicates the ratio of DEGs enriched in this pathway to the total number of papaya predict genes in this pathway.

For the global functional analysis of DEGs, GO annotation was performed using Blast2GO. Out of the 842 DEGs, 507 corresponding proteins were associated with at least one GO term. The up- and down- regulated DEGs annotated in GO were grouped into 45 and 37 groups based on GO level2 classification, respectively (Figure 3A, Figure S2, and Tables S1A,B). The assigned GO terms belonged to three main ontologies: molecular function, biological process and cellular component. In the molecular function, the most common categories were “catalytic activity” (154 up-regulated, 135 down-regulated) and “binding” (123 and 125, respectively), followed by “transporter activity” and “transcription factor activity.” In the category of cellular component ontology, membrane and cell acted as two primary places. Within biological process, “metabolic process,” “cellular process,” and “single-organism process” were predominant. We further applied GO category enrichment analysis to elucidate the functional enrichment of the DEGs, using Fisher's exact test with an FDR cutoff ≤ 0.05. A total of 45 GO terms were enriched in biological processes, molecular function and cellular components (See Table S2, Figure S3). The biological process GO term “microtubule-based movement” was the most significantly and specifically overrepresented term, followed by “polysaccharide catabolic process,” “sucrose metabolic process,” and “cell wall organization or biogenesis.” The enrichment in the biological process category was also reflected by enrichment in the molecular function GO terms “microtubule motor activity,” “microtubule binding,” and “tubulin binding” and by enrichment in the cellular component GO terms “extracellular region,” “cell wall,” “external encapsulating structure,” and “microtubule cytoskeleton.”

All detected DEGs were blasted to STRING 9.0 for further annotation based on Cluster of Orthologous Groups (COG) of protein categories. A total of 161 differentially expressed genes with at least one COG annotations were grouped into 22 of 25 functional categories (Figure S4). The largest category was “General function prediction only” (36 COG annotations), followed by “Signal transduction mechanisms” (20), “Cytoskeleton” (16) and “Secondary metabolites biosynthesis, transport and catabolism” (16). Only 7 COG annotations belonged to the “Function unknown.”

In addition, prediction of the biochemical pathways associated with the DEGs was performed by the Kyoto Encyclopedia of Genes and Genomes (KEGG) identifiers. We used hypergeometric test to identify those pathways that were significantly affected using an FDR ≤ 0.05, relative to the whole papaya transcriptome background. Of the 842 DEGs, 95 genes were assigned at least a KO ID per gene and categorized into 125 pathways. Seven pathways, phenylpropanoid biosynthesis (PATHWAY: ko00940), cutin, suberine, and wax biosynthesis (PATHWAY: ko00073), starch and sucrose metabolism (PATHWAY: ko00500), chemical carcinogenesis (PATHWAY: ko05204), pentose and glucuronate interconversions (PATHWAY: ko00040), drug metabolism-cytochrome P450 (ko00982) and metabolism of xenobiotics by cytochrome P450 (ko00980) were significantly overrepresented with corrected *p*-values smaller than 0.05 among the differentially expressed genes between two cultivars (Figure 3B, Table S3). However, for the FDR threshold less than 0.01 no significant KEGG pathways have been detected.

### DEGs related to transcription factors, transporter proteins and hormones

**Figure 4:**
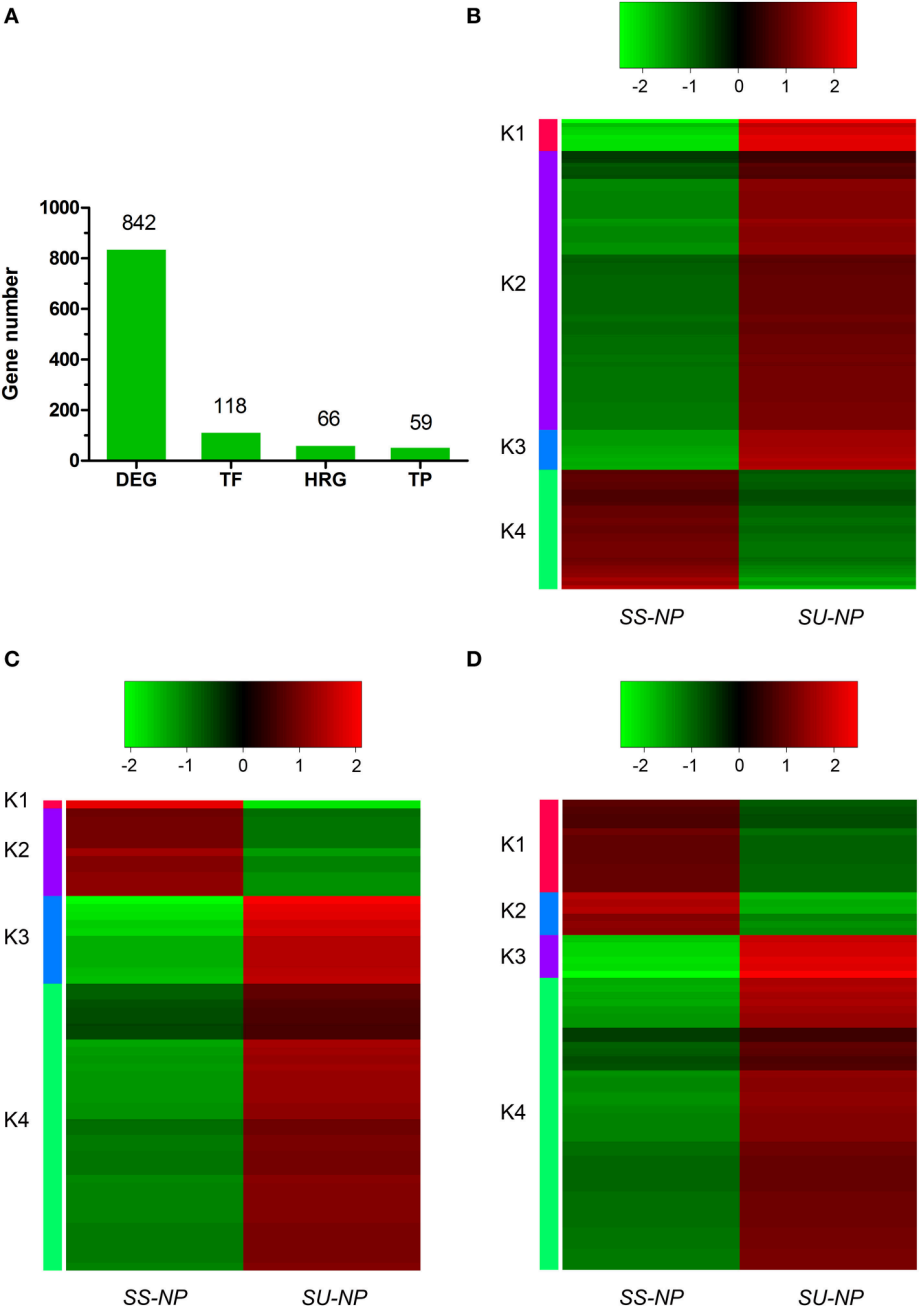
**Differentially expressed gene numbers and expression patterns of transcription factors (TFs), transporter proteins (TPs) and hormone-related genes (HRGs). (A)** Numbers of total DEGs and those annotated as TFs, TPs and HRGs. **(B–D)** Heat maps showing log_2_ FPKM values of 118 TFs, 59 TPs and 66 HRGs annotated DEGs.

Expression profiles of the DEGs classified as genes encoding transcription factors (TFs), transporter proteins (TPs) and hormone-related genes (HRGs) between non-GE and GE cultivars were further analyzed on the basis of annotations in *Arabidopsis thaliana*. In total, we identified 118, 59 and 66 DEGs encoding TFs, TPs and HRGs (Figure 4, Tables S4–S6). Heat map results indicated that most of the DEGs between two cultivars, or between each of the four clusters, showed distinct expression dynamics. Most DEGs belonging to these three types were up-regulated in the transgenic cultivar.

Numerous transcription factor families, including *AP2/ERF* (ethylene response factor), *MYB, WRKY, bZIP, bHLH, NAC, DREB*, kinase and C2H2-type Zn-finger, were identified to be induced or suppressed upon introduction of the PRSV CP transgene in this study (Figure 4B). Of the 118 TFs, 88 genes were up-regulated and 30 were down-regulated in response to the introduction of the PRSV CP transgene. There were 115 TFs expressed in both cultivars, but one TF (evm.TU.supercontig_55.62) was specifically expressed in SunUp and two TFs (evm.TU.supercontig_19.100 and evm.TU.supercontig_14.16) were specifically expressed in Sunset (Table S4). These three genes were all annotated as basic helix-loop-helix (*bHLH*) DNA-binding superfamily protein in *Arabidopsis thaliana*. In terms of the most abundantly differentially expressed TFs (FPKM≥2, absolute fold change >2.5, FDR < 0.01), a total of 35 genes encoding TFs in *Arabidopsis* were identified, of which 33 were up-regulated in SunUp and only 2 down-regulated (Table 3).

**Table 3:** **The most abundantly differentially expressed transcription factor (TF) genes between two cultivars**.

**Gene ID**	***Arabidopsis***	**Gene annotation**	**Expression level (FPKM)**		**Cluster**
		**TFs**	**SS-NP**	**SU-NP**	
evm.TU.supercontig_58.37	AT5G43650.1	Basic helix-loop-helix (bHLH) DNA-binding superfamily protein	6.27135	223.272	K1
evm.TU.supercontig_807.3	AT5G13080.1	WRKY DNA-binding protein 75	2.86936	63.0173	K1
evm.TU.supercontig_50.27	AT3G23230.1	Integrase-type DNA-binding superfamily protein	3.81205	79.7241	K1
evm.TU.supercontig_26.302	AT4G12020.2	Protein kinase family protein	2.3915	77.3382	K1
evm.TU.supercontig_190.29	AT2G28710.1	C2H2-type zinc finger family protein	4.24345	109.473	K1
evm.TU.supercontig_34.60	AT1G34670.1	Myb domain protein 93	26.4289	555.113	K1
evm.TU.supercontig_126.44	AT5G26170.1	WRKY DNA-binding protein 50	3.85459	99.2079	K1
evm.TU.supercontig_74.83	AT4G25480.1	Dehydration response element B1A	11.6146	75.5762	K2
evm.TU.supercontig_34.213	AT4G12020.2	Protein kinase family protein	21.0464	131.526	K2
evm.TU.supercontig_152.35	AT1G62300.1	WRKY family transcription factor	22.0205	136.961	K2
evm.TU.supercontig_9.35	AT5G49520.1	WRKY DNA-binding protein 48	17.2988	106.025	K2
evm.TU.supercontig_14.99	AT5G61430.1	NAC domain containing protein 100	6.85613	43.7501	K2
evm.TU.supercontig_21.63	AT1G71520.1	Integrase-type DNA-binding superfamily protein	9.59186	59.684	K2
evm.TU.supercontig_3.487	AT4G12020.2	Protein kinase family protein	67.3238	442.734	K2
evm.TU.supercontig_129.23	AT1G30330.2	Auxin response factor 6	49.3207	288.859	K2
evm.TU.supercontig_95.50	AT4G12020.3	Protein kinase family protein	16.5506	126.303	K2
evm.TU.supercontig_3.494	AT1G68320.1	Myb domain protein 62	23.1542	170.373	K2
evm.TU.supercontig_3.54	AT4G23810.1	WRKY family transcription factor	2.49472	19.0917	K2
evm.TU.supercontig_27.16	AT4G12020.1	Protein kinase family protein	4.49573	34.731	K2
evm.TU.supercontig_20.220	AT4G12020.3	Protein kinase family protein	18.0854	120.054	K2
evm.TU.supercontig_12.9	AT4G12020.2	Protein kinase family protein	3.27164	26.05	K2
evm.TU.supercontig_594.1	AT5G18270.1	NAC domain containing protein 87	7.7607	57.9356	K2
evm.TU.supercontig_104.8	AT5G06510.2	Nuclear factor Y, subunit A10	2.45138	22.29	K2
evm.TU.supercontig_111.6	AT3G47600.1	Myb domain protein 94	2.70301	15.5779	K2
evm.TU.supercontig_11.111	AT2G38250.1	Homeodomain-like superfamily protein	2.83535	18.2849	K2
evm.TU.supercontig_578.2	AT4G12020.2	Protein kinase family protein	15.3704	137.273	K3
evm.TU.supercontig_96.45	AT2G28500.1	LOB domain-containing protein 11	6.45897	62.6016	K3
evm.TU.supercontig_145.21	AT2G47190.1	Myb domain protein 2	41.5383	363.428	K3
evm.TU.supercontig_67.57	AT3G12910.1	NAC (No Apical Meristem) domain transcriptional regulator superfamily protein	2.19042	28.8537	K3
evm.TU.supercontig_5.242	AT2G38470.1	WRKY DNA-binding protein 33	44.8722	421.063	K3
evm.TU.supercontig_101.16	AT2G44840.1	Ethylene-responsive element binding factor 13	12.759	131.366	K3
evm.TU.supercontig_50.56	AT5G45710.1	Winged-helix DNA-binding transcription factor family protein	7.74852	96.4934	K3
evm.TU.supercontig_70.106	AT5G14000.1	NAC domain containing protein 84	46.7068	489.497	K3
evm.TU.supercontig_29.134	AT2G29660.1	Zinc finger (C2H2 type) family protein	18.3347	3.17347	K4
evm.TU.supercontig_209.9	AT3G58120.1	Basic-leucine zipper (bZIP) transcription factor family protein	15.1098	2.47565	K4

*Genes were filtered by (1) FPKM≥2 in each libraries, (2) absolute fold change >2.5 and an FDR significance score < 0.01 for each gene between two cultivars*.

Hierarchical clustering of 59 differentially expressed TPs showed extensive differences between Sunset and SunUp. Among these DEGs, 47 were found to be up-regulated while only 12 were down-regulated in SunUp (Figure 4C, Table S5). We found that genes annotated as ABC-2 type transporter, tonoplast intrinsic protein, laccase 11, and plant L-ascorbate oxidase in *Arabidopsis thaliana* were down-regulated in SunUp, while genes such as nitrate transporter, zinc transporter, mechanosensitive channel of small conductance-like were significantly up-regulated in SunUp (Table 4).

**Table 4:** **The most abundantly differentially expressed transporter (TP) genes between two cultivars**.

**Gene ID**	***Arabidopsis***	**Gene annotation**	**Expression level (FPKM)**		**Cluster**
		**TPs**	**SS-NP**	**SU-NP**	
evm.TU.supercontig_3.5	AT5G52860	ABC-2 type transporter family protein	45.0057	2.33714	K1
evm.TU.supercontig_190.36	AT4G01470	Tonoplast intrinsic protein 1;3	139.781	22.6553	K2
evm.TU.supercontig_26.308	AT5G03260	Laccase 11	22.893	3.84154	K2
evm.TU.supercontig_103.53	AT5G21105	Plant L-ascorbate oxidase	15.2722	2.12329	K2
evm.TU.supercontig_69.87	AT1G12940	Nitrate transporter2.5	2.67323	66.8621	K3
evm.TU.supercontig_307.1	AT3G12750	Zinc transporter 1 precursor	3.58291	62.3305	K3
evm.TU.supercontig_163.8	AT2G38100	Proton-dependent oligopeptide transport (POT) family protein	26.399	356.769	K3
evm.TU.supercontig_6.348	AT2G17500	Auxin efflux carrier family protein	18.3344	199.704	K3
evm.TU.contig_36671.1	AT5G12380	Annexin 8	7.06387	90.9406	K3
evm.TU.supercontig_97.97	AT3G13080	Multidrug resistance-associated protein 3	2.18211	23.0908	K3
evm.TU.supercontig_5.274	AT1G69870	Nitrate transporter 1.7	25.9909	203.961	K3
evm.TU.supercontig_96.2	AT5G53130	Cyclic nucleotide gated channel 1	11.6095	93.1071	K3
evm.TU.supercontig_1109.2	AT1G12600	UDP-N-acetylglucosamine (UAA) transporter family	2.04974	24.1666	K3
evm.TU.contig_35659.1	AT4G01010	Cyclic nucleotide-gated channel 13	5.86577	60.6668	K3
evm.TU.supercontig_28.77	AT5G12080	Mechanosensitive channel of small conductance-like 10	9.12271	61.9686	K4
evm.TU.supercontig_30.98	AT3G12750	Zinc transporter 1 precursor	3.50902	25.8696	K4
evm.TU.supercontig_19.10	AT1G66950	Pleiotropic drug resistance 11	7.24251	46.3446	K4
evm.TU.supercontig_10.181	AT1G15460	HCO3- transporter family	2.88164	21.3624	K4
evm.TU.supercontig_33.12	AT1G23300	MATE efflux family protein	21.1118	122.4	K4
evm.TU.supercontig_19.9	AT2G36380	Pleiotropic drug resistance 6	4.99586	32.2541	K4
evm.TU.contig_31100.1	AT1G08920	ERD (early response to dehydration) six-like 1	2.54556	17.2646	K4

*Genes were filtered by (1) FPKM≥2 in each libraries, (2) absolute fold change >2.5 and an FDR significance score < 0.01 for each gene between two cultivars*.

With respect to hormone-related genes, 66 of these genes were differentially regulated between Sunset and SunUp, consisting of 47 up-regulated and 19 down-regulated DEGs (Figure 4D, Table S6). Regarding the most abundantly differentially expressed hormone-related genes, there were only 3 down-regulated genes consisting of 2 abscisic acid (ABA) related genes and 1 gibberellin related gene (Table 5). By contrast, 22 genes were found to be up-regulated in SunUp, including 10 ABA, 9 salicylic acid (SA), 6 ethylene, 5 auxin, 1 JA and 1 brassinosteroid HRGs. Interestingly, two auxin-responsive genes (evm.TU.supercontig_37.203, evm.TU.supercontig_37.215) were found to be only expressed in Sunset, whereas two gibberellin-related genes (evm.TU.contig_47211.1, evm.TU.supercontig_133.24) were exclusively expressed in SunUp (Table S6).

**Table 5:** **The most abundantly differentially expressed hormone-related genes between two cultivars**.

**Gene ID**	***Arabidopsis***	**Gene annotation**	**Related Hormone**	**Expression level (FPKM)**		**Cluster**
				**SS-NP**	**SU-NP**	
evm.TU.supercontig_27.36	AT2G33790	Arabinogalactan protein 30	Abscisic acid	126.905	10.0667	K2
evm.TU.supercontig_14.88	AT5G14920	Gibberellin-regulated family protein	Gibberellin	33.3247	2.15195	K2
evm.TU.supercontig_41.42	AT1G02205	Fatty acid hydroxylase superfamily	Abscisic acid	184.548	24.8403	K2
evm.TU.supercontig_157.25	AT5G59310	Lipid transfer protein 4	Abscisic acid	83.6708	1225.39	K3
evm.TU.supercontig_50.27	AT3G23230	Integrase-type DNA-binding superfamily protein	Ethylene	3.81205	79.7241	K3
evm.TU.supercontig_157.27	AT5G59310	Lipid transfer protein 4	Abscisic acid	23.3339	433.616	K3
evm.TU.supercontig_34.60	AT1G34670	Myb domain protein 93	Abscisic acid auxin salicylic acid	26.4289	555.113	K3
evm.TU.supercontig_6.348	AT2G17500	Auxin efflux carrier family protein	Auxin	18.3344	199.704	K4
evm.TU.supercontig_50.56	AT5G45710	Winged-helix DNA-binding transcription factor family protein	Auxin ethylene	7.74852	96.4934	K4
evm.TU.supercontig_5.242	AT2G38470	WRKY DNA-binding protein 33	Abscisic acid	44.8722	421.063	K4
evm.TU.supercontig_101.16	AT2G44840	Ethylene-responsive element binding factor 13	Ethylene	12.759	131.366	K4
evm.TU.supercontig_145.21	AT2G47190	Myb domain protein 2	Abscisic acid ethylene salicylic acid	41.5383	363.428	K4
evm.TU.supercontig_23.98	AT3G24500	Multiprotein bridging factor 1C	Ethylene abscisic acid	21.5776	176.415	K4
evm.TU.contig_34243.1	AT2G29420	Glutathione S-transferase tau 7	Salicylic acid	29.2826	234.884	K4
evm.TU.supercontig_3.494	AT1G68320	Myb domain protein 62	Salicylic acid	23.1542	170.373	K4
evm.TU.supercontig_48.133	AT5G26920	Cam-binding protein 60-like G	Salicylic acid	49.9857	327.068	K4
evm.TU.supercontig_3.54	AT4G23810	WRKY family transcription factor	Salicylic acid	67.3238	442.734	K4
evm.TU.supercontig_59.60	AT2G29420	Glutathione S-transferase tau 7	Salicylic acid	29.6253	202.733	K4
evm.TU.supercontig_2.408	AT2G30020	Protein phosphatase 2C family protein	Abscisic acid	59.7727	403.526	K4
evm.TU.supercontig_74.83	AT4G25480	Dehydration response element B1A	Salicylic acid	11.6146	75.5762	K4
evm.TU.supercontig_3.487	AT4G21410	Cysteine-rich RLK (RECEPTOR-like protein kinase) 29	Abscisic acid	2.49472	19.0917	K4
evm.TU.supercontig_129.23	AT5G65670	Indole-3-acetic acid inducible 9	Auxin	49.3207	288.859	K4
evm.TU.supercontig_14.99	AT5G61430	NAC domain containing protein 100	Brassinosteroid	6.85613	43.7501	K4
evm.TU.supercontig_111.6	AT3G47600	Myb domain protein 94	Abscisic acid auxin ethylene jasmonic acid salicylic acid	2.70301	15.5779	K4
evm.TU.contig_31100.1	AT1G08920	ERD (early response to dehydration) six-like 1	Abscisic acid	2.54556	17.2646	K4

*Genes were filtered by (1) FPKM≥2 in each libraries, (2) absolute fold change >2.5 and an FDR significance score < 0.01 for each gene between two cultivars*.

### Confirmation of candidate DEGs by qRT-PCR analysis

**Figure 5:**
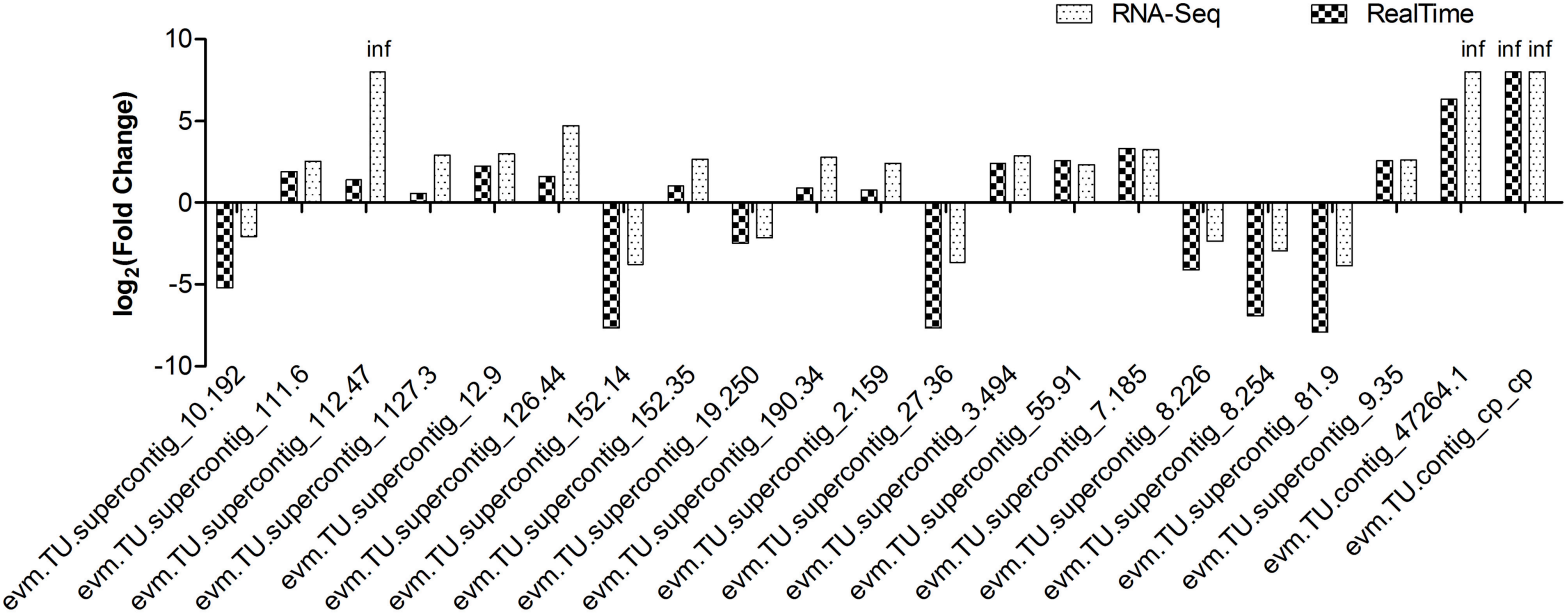
**Expression pattern validation of 21 selected DEGs by qRT-PCR in transgenic SunUp relative to the donor control Sunset**. The transcriptional level of candidate genes was examined by real-time PCR with three biological replicates. *EIF* was used as an internal control. RNA-Seq data were highly consistent with qRT-PCR results (*r* = 0.9247). The *Y*-axis indicates the fold change of transcript abundance in SU-NP relative to the control SS-NP.

To confirm the accuracy of Illumina RNA-Seq results, a subset of 21 genes, which were differentially expressed between non-GE and GE papaya, was chosen for quantitative real-time PCR analysis. The primer sequences, gene annotations, and FPKM and qRT-PCR expression values were listed in Tables S7–S9. Although the fold-changes in transcript abundance determined by RNA-Seq and qRT-PCR did not exactly match, all of the 21 qRT-PCR analyses showed trends of expression (up- or down-regulation) similar to those revealed by RNA-Seq results (Figure 5). The expression patterns from the two platforms were largely consistent, with a Pearson's correlation coefficient of 0.9247.

### Alternative splicing of transcripts in the two papaya cultivars

**Figure 6:**
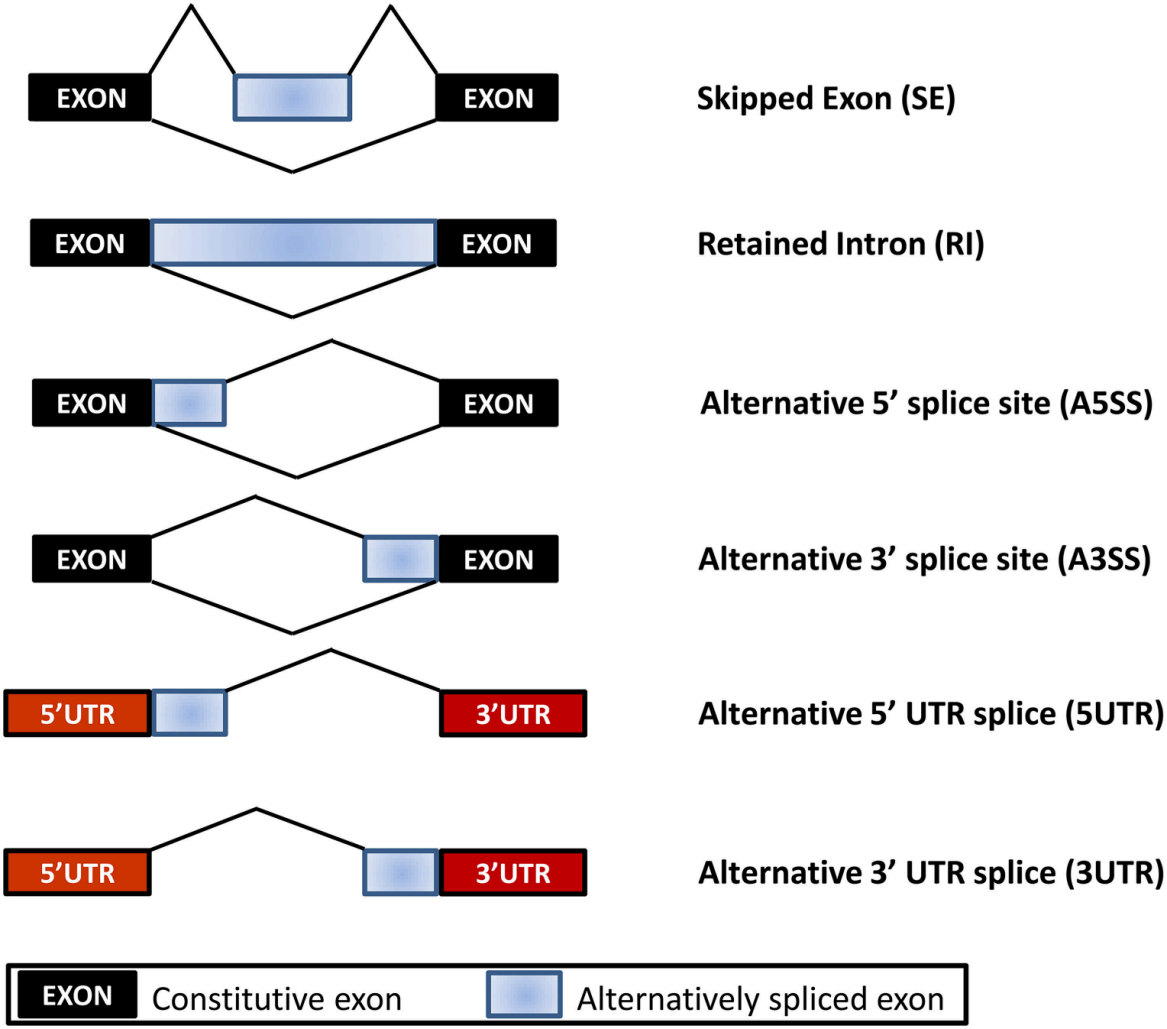
**Types of Alternative Splicing Events**. Skipped Exon (SE): one or more exons are spliced out of the mature messenger RNA (mRNA). Retained Intron (RI): one or more introns are included in the mature mRNA; Alternative 5′ (or 3′) splice site (A5SS or A3SS): distinct 5′ (or 3′) splice site corresponds to a longer or shorter exon, which are particularly difficult to interrogate by microarray analysis due to the small variably included region; 5UTR or 3UTR: alternative promoter use results in shorter or longer 5′ UTR or 3′ UTR isoforms.

**Figure 7:**
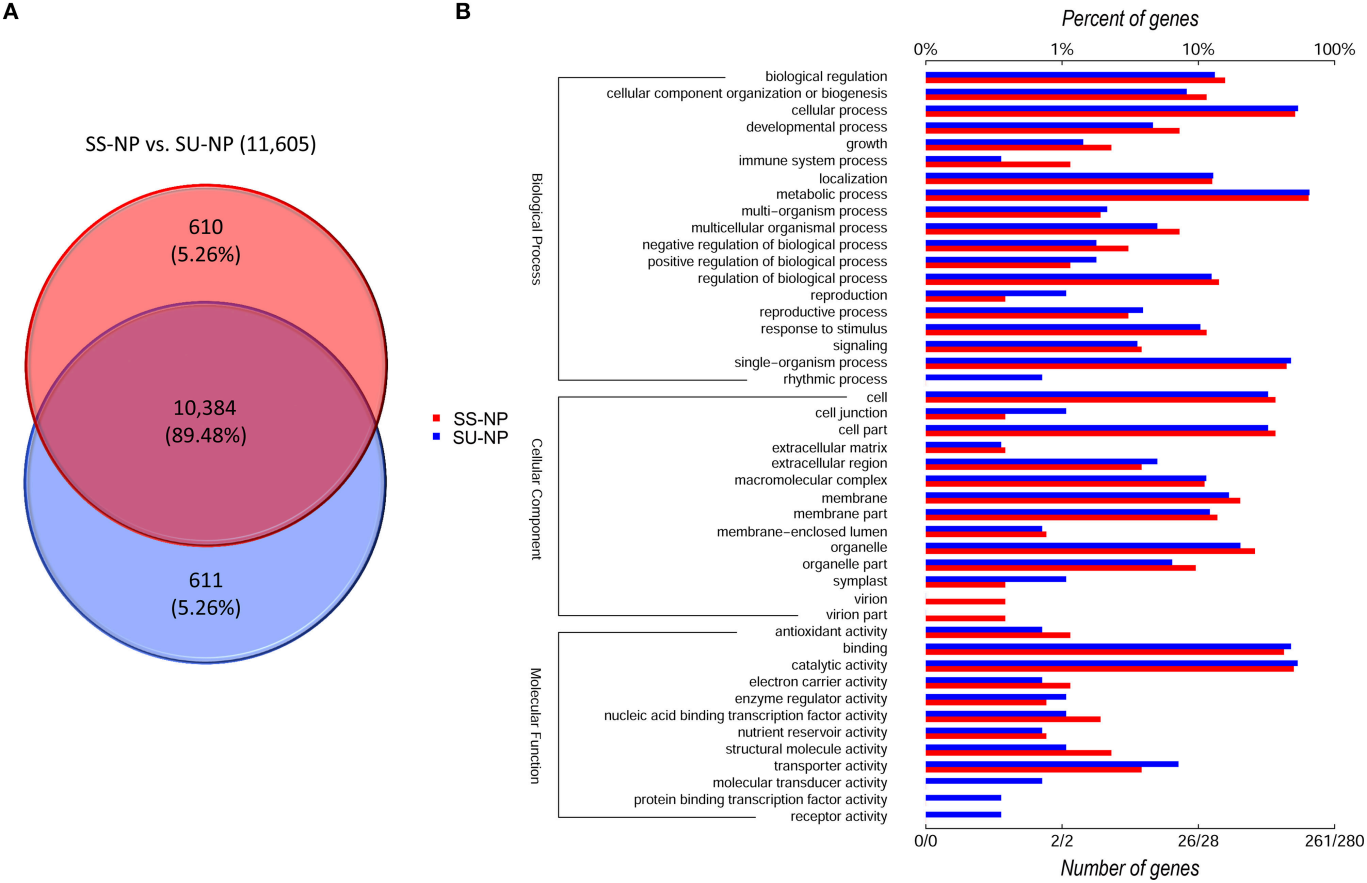
**Venn diagram and functional enrichment analysis of genes showing AS events between PRSV-negative Sunset and SunUp. (A)** Unique and shared genes between two varieties. **(B)** Level 2 GO annotation of variety-specific genes undergoing AS events comparison between Sunset and SunUp. We divided the sets into three major GO ontologies: biological process, cellular component and molecular function. Red and blue bars represent the number and percentage of genes specific in Sunset and SunUp, respectively.

To investigate the patterns of AS in non-GE and GE papaya, we developed a series of in-house Perl scripts that could detect the variations in the splicing structure and identify AS events by extracting information from the output of TopHat. In this study, we focused on six types of AS events: SE, RI, A5SS, A3SS, 5UTR, and 3UTR (Figure 6, Table 6), by comparing transcripts with the papaya annotated gene model. Among the detected AS events, SE was the most abundant type (53%), followed by A3SS (17%), A5SS (10%), 3UTR (10%), 5UTR (7%), and RI (3%) (Table 6). In total, we identified 67,686 AS events in Sunset and 68,455 AS events in SunUp. However, the number of genes exhibiting AS events in both cultivars was similar, mapping to 10,994 and 10,995 papaya annotated genes, respectively. Around 610 genes were specifically alternatively spliced in Sunset and 611 genes in SunUp (Figure 7A).

**Table 6:** **Summary of the six types of alternative splicing events of papaya Sunset and SunUp**.

**Type of event**	**SS-NP**		**SU-NP**		**Different genes between SS-NP and SU-NP**
	**events**	**Genes**	**Events**	**Genes**	
SE	36,167 (53.4%)	7139	36,075 (52.7%)	7136	263
RI	1722 (2.5%)	1154	1707 (2.5%)	1149	696
A5SS	7024 (10.4%)	3400	7,188 (10.5%)	3370	855
A3SS	11,409 (16.9%)	4186	11,825 (17.3%)	4221	803
5UTR	4393 (6.5%)	2198	4683 (6.8%)	2221	645
3UTR	6971 (10.3%)	3962	6,977 (10.2%)	3945	924
Total	67,686	10,994	68,455	10,995	1221

*SE, skipped exon; RI, retained intron; A5SS, alternative 5′ splice site; A3SS, alternative 3′ splice site; 5UTR, alternative 5′ UTR splice; 3UTR, alternative 3′ UTR splice*.

We further classified those genes that underwent AS events on the basis of gene ontology annotation into three component ontologies: molecular function (MF), biological process (BP) and cellular component (CC) (Figure 7B, Tables S10A,B). In both cultivars, the molecular function ontology included a high portion of catalytic activity (50.19%, 53.57%) and protein binding (42.53%, 47.86%). With regard to the cellular component, 73.56% (Sunset) and 65% (SunUp) were assigned to cell or cell part followed by membrane, and organelle. In the biological process ontology, metabolic process (64.37%, 65.36%), cellular process (51.34%, 53.93%) and single-organism process (44.44%, 47.86) were the top three most dominant groups. It is noteworthy that some GO annotation for the genes displaying AS events exclusively in Sunset was significantly different from that in SunUp. For instance, together 0.77% (evm.TU.supercontig_4800.1) was assigned to virion or virion part in Sunset while no corresponding GO terms were assigned in SunUp. Genes involved in immune system process (BP), structural molecule activity (MF) and nucleic acid binding transcription factor activity (MF) in Sunset were much more abundant than those in SunUp. Conversely, compared to assigned GO terms in Sunset, four additional annotations were found in SunUp: protein binding transcription factor activity, molecular transducer activity, and receptor activity in category of molecular function, and rhythmic process in biological process. Terms such as reproduction (BP), cell junction (CC), symplast (CC), and transporter activity (MF) enriched a much higher percentage of genes in SunUp than that in its progenitor cultivar Sunset.

## Discussion

The primary objective of this study was to identify genome-wide perturbations of steady state levels of transcripts in young healthy leaves of transgenic papaya subjected to bombardment and expression of the PRSV CP transgene. Here, we carried out paired end sequencing of RNA-Seq libraries prepared from mRNA isolated from 4-month old leaves of SunUp and its progenitor Sunset grown under the same conditions prior to infection with PRSV. High throughput sequencing generated more than 342 million filtered reads and nearly 80% of the total reads were uniquely mapped to the papaya reference genome. Subsequently, resultant transcriptome of unique reads were used in the downstream analysis of expression (FPKM values), gene ontologies and other functional categories. Results showed that the expression profiles of multiple genes are altered in transgenic cultivar SunUp and these transcripts are components of important disease resistant processes, hormonal signaling pathways and regulatory networks. The detected expression patterns from two platforms, RNA-Seq and qRT-PCR, were highly consistent (Figure 5), confirming the accuracy and reliability of the RNA-Seq results.

### Several factors responsible for variable gene expression after transgene insertion

RNA-Seq expression profiles in SunUp and Sunset were similar, consistently with previous RNA-Seq studies, showing that gene expression follows a bimodal distribution of high and low expression genes (Hebenstreit et al., 2011). Studies have demonstrated that highly expressed genes are active genes performing the necessary cellular processes while other genes are likely to be the by-products of biological or experimental noise (Hebenstreit et al., 2011; Hart et al., 2013). Approximately 94% of mapped genes were shared by the transcriptomes of Sunset and SunUp with other 620 and 662 specifically expressed genes, respectively (Figure 1D). After strict filtering criteria, out of all mapped genes, only 842 DEGs were identified, 475 of which were up-regulated in SunUp and 367 down-regulated (Figure 2). Several factors were responsible for the identified variations in gene expression between transgenic cultivar SunUp and its progenitor Sunset.

The introduction of PRSV CP transgene and other unexpected DNA fragments was considered to be a major causal effect of differential gene expression. The faithful execution of *cp* gene expression might require a precise and carefully orchestrated set of steps that depend on the proper cooperation of the molecular machinery (general transcription factors, activators and coactivators). On the other hand, transgene integration position within the genome is associated with variable gene expression (Matzke and Matzke, 1998). The insertions of the exogenous gene or the unintended DNA fragments could probably cause a mutation and altered expression of the gene into which it inserts. Or the insertions might muck up the various classes of transcriptional regulatory elements adjacent to the genes that they regulate, such as core/proximal promoters, distal enhancers, silencers, insulators/boundary elements, and locus control regions (LCR) (Maston et al., 2006), hence affecting the controlled patterns of gene expression. Three transgenic insertions with flanking border sequences were able to be identified from corresponding (bacterial artificial chromosome) BAC clones or sequences reads from the whole-genome shotgun (WGS) sequence of SunUp papaya, and the number and types of inserts were subsequently confirmed by Southern analysis using probes spanning the entire transformation plasmid (Suzuki et al., 2008). However, the organelle-like borders of three transgenic insertions make them difficult to be assembled and located into SunUp reference genome because nuclear organelle DNA (norgDNA) are abundance in nuclear genomes (Martin et al., 2002; Shahmuradov et al., 2003). In present study, the lack of information on the precise location of the integrated region kept us from thorough and systematical examination of their impact on the function of interrupted genes and neighboring genes. From this point of view, a completely sequenced papaya genome in the near future will complement the insufficiency of the present study. In addition to these three large insertions, multiple copies of the three insertions or some other small insertions of a few base pairs (< 20 bp) induced by particle bombardment may exist but they are below the detection limit of PCR and Southern blot analysis which are the standard methods used to detect integration of vector sequences (Endo et al., 2015). Increased transgene copy number can result in higher or lower expression level (Sijen et al., 1996; Wang and Waterhouse, 2000; Altpeter et al., 2005), and the existence of small undetected exogenous sequences may also have some profound effects on gene expression levels and/or protein function.

Besides transgene copy numbers and integration position effects, other factors such as somaclonal variations (Phillips et al., 1994; Duncan, 1996; Kaeppler et al., 2000) and spontaneous mutations during meiosis (Magni, 1963; Golubovskaya et al., 1992; Lercher and Hurst, 2002) of over 20 generations might induce segregated SNPs, InDels, and rearrangement, which would lead to the divergence of gene expression between GE and non-GE papaya cultivars. Additionally, sources of bias and error, such as technical variability during library preparation and sequencing (Hansen et al., 2010; Roberts et al., 2011), biological variability between replicates of the same sample (Trapnell et al., 2012), and the inevitable error rates during reconstruction of all full-length transcripts from short reads (Li et al., 2010; Grabherr et al., 2011), could also account for this difference. The random distribution of DEGs amongst papaya chromosomes (Table 2) suggests that bombardment-mediated transformation might have a genome-wide effect on the papaya genome, not just specifically affecting the flanking sequences of insertion sites.

### Enrichment of disease resistance genes in DEGs

With respect to gene ontologies, 10 GO terms were assigned exclusively to down-regulated genes and two terms to up-regulated genes (Figure 3A, and Tables S1A,B). This finding revealed that unique cellular functions have been created in the transgenic cultivar in acquiring resistance to PRSV, and these two cultivars could already have developed different genetic pathways for adaptation to the environment even at an early growth stage. For instance, the up-regulated gene in the SunUp genome associated with the “virion part” or “virion” was found to be a coat protein (*cp*) gene, which was the target gene derived from PRSV HA 5-1 that was bombarded into the papaya genome using gene gun DNA particle bombardment. Previous studies on virus-derived resistance in transgenic tobacco plants revealed that transgenic tobacco plants expressed high levels of the TMV CP and were more resistant to TMV virions than to TMV RNA inoculations (Abel et al., 1986). CP-mediated protection (CPMP) against TMV occurs through the inhibition of virion disassembly in the originally infected cells (Register and Beachy, 1988). There is limited information regarding the developmental stages where a transgenic plant is capable of performing RNA silencing. An intriguing finding has shown that high levels of transgene-derived short interfering RNAs (siRNAs), the key constituents in PTGS/RNAi triggering for the process which leads to degradation of homologous RNAs (Hutvágner and Zamore, 2002), were generated in mature transgenic potato plants (*Solanum tuberosum* L.) (at least 2 months old) prior to potato virus Y (PVY) inoculation, and the concentration further increased as the plants grew (Missiou et al., 2004). We used 4-month-old plants in this study, allowing sufficient time for the siRNAs to be generated and have initiated biological processes that differed from those in non-transgenic plants. Three up-regulated genes, evm.TU.supercontig_120.27, evm.TU.supercontig_1.397, and evm.TU.supercontig_48.133, were involved exclusively in the assigned term “immune system process” in SunUp transcriptomes. Their functions are ammonium transporter 2 (AMT2), protein of unknown function (DUF506), and calmodulin-binding protein 60-like (CBP60g) annotated in *Arabidopsis* and Pfam database, respectively (data not shown), suggesting those genes play roles in defending the PRSV disease. CBP60g, which belongs to a new family of transcription factors triggered by microbe-associated molecular patterns (Vlot et al., 2009; Wang et al., 2009; Wan et al., 2012) was found in many studies to be a positive regulator of disease resistance via the SA signaling pathway. GO enrichment analysis reflected that microtubule-related categories were highly enriched among these DEGs, followed by polysaccharide catabolic and sucrose metabolic processes (Table S2). These enriched processes mostly take place in the “extracellular region” and “cell wall.” Microtubules are one of the major components of the cytoskeleton, and found to carry out a wide range of functions in structural support, intracellular transport, cell motility and DNA segregation and other fundamental biological functions (Vale, 1987; Mandelkow and Mandelkow, 1995; Nogales, 2001; Wittmann and Waterman-Storer, 2001; Garner et al., 2004). Microtubules often interact with three main classes: the microtubule-associated proteins (MAPs), the motor proteins and proteins that are not normally called MAPs but often found associated with microtubules and may even copurify with them (Mandelkow and Mandelkow, 1995). Representatives are MAP1a and 1b, MAP2a, 2b, and 2c, MAP4, tau protein, kinesin, dynein, glycolytic enzymes and kinases. The finding of enriched microtubule-related categories prompted a hypothesis that microtubule-associated proteins (MAPs), motor proteins and other related proteins are activated in response to the structural changes caused by particle bombardment to stabilize cellular structures. Numerous genetic studies have provided new lines of evidence implicating that cell wall polysaccharides function as latent signal molecules during pathogen infection and elicit defense responses by the plant (Vorwerk et al., 2004). A report has also revealed that sucrose accumulated at higher levels in leaves of disease resistance transgenic rice (Oryza sativa L.), and pretreatment of rice plants with sucrose was able to enhance resistance to M. *oryzae* infection, supporting a sucrose-mediated priming of defense responses in transgenic rice which results in broad-spectrum protection against pathogens (Gómez-Ariza et al., 2007). The enrichment in GO terms “polysaccharide catabolic process” and “sucrose metabolic process” suggested this potential relationship between disease resistance and sucrose metabolism in the transgenic cultivar SunUp.

Based on the KEGG enrichment analysis, no significant pathways were detected with the FDR threshold less than 0.01, indicating that the transgenic cultivar SunUp did not undergo major changes upon DNA particle bombardment. Whereas seven pathways including phenylpropanoid biosynthesis, cutin, suberine and wax biosynthesis, and starch and sucrose metabolism and others were overrepresented for the FDR cut-off of 0.05 (Figure 3B, Table S3). Some previous observations provided direct evidence that natural products synthesized by plants such as phenylpropanoid products contribute to their resistance to pathogens, and this increased resistance is a pathogen-induced response but dependent on developmental accumulation of phenylpropanoid products (Maher et al., 1994). Hence, the overrepresented pathway “phenylpropanoid biosynthesis” pathway could be explained by the increased disease susceptibility of non-transgenic cultivar via synthesizing lower levels of phenylpropanoid products during development compared with its transgenic cultivar.

### The key regulons (TFs, TPs, and HRGs) induce defense response

The activities of transporters and transcription factors are the two main categories in the molecular function based on GO analysis of DEGs. The increased vigor and growth and the nature of high resistance to PRSV in SunUp indicate that SunUp and Sunset cultivars must sense and respond to their environment differently in a complex manner, through signaling and regulatory pathways mediated by phytohormones such as jasmonic acid (JA) and salicylic acid, generally resulting in altered expression of transcription factors and in enhanced or repressed expression of genes encoding related proteins. Hence, the expression patterns of TFs, TPs, and HRGs were dissected in this study to detect their changes in expression after bombardment transformation and under years of the continuous challenge of virus inoculations by the natural aphid vector.

The highly variable expression patterns of TFs, TPs, and HRGs reflected the significant changes of expression that occurred upon transgene insertion and that may play critical roles in plant resistance to pathogens. Transcription factors, interacting with the transcriptional regulatory elements (enhancers, promoters, silencers, insulators, and LCR regions) adjacent to the genes that they regulate, have the ability to control the expression of many downstream genes to regulate diverse biological processes. A slight alteration in transcript abundance of TFs can trigger a cascade of reactions implicated in many physiological processes resulting in a substantial change in downstream gene expression (Ramirez and Basu, 2009; Wang et al., 2013b). Hence, the transcriptional level of the gene is either up- or down-regulated depending on the adjacent transcription factor. Numerous transcription factor families were detected to be induced or suppressed after transgene insertion (Figure 4B). Several families of the TFs, such as *MYB, WRKY, ERF, NAC*, kinase and zinc-finger, which are known key regulators in the defense response (Xie et al., 1999; Gutterson and Reuber, 2004; Liu et al., 2004; Ryu et al., 2006; Eulgem and Somssich, 2007; Guo et al., 2009; Nuruzzaman et al., 2010, 2013), were most highly expressed in transgenic papaya (Table 3). Silencing of genes encoding the mitogen-activated protein kinase (MAPK) *NTF6* or the MAPK kinase *MEK1* attenuated tobacco *N*-mediated resistance to TMV, where the defense response *NbWRKY1-NbWRKY3* and *NbMYB1* transcription factors are also involved (Liu et al., 2004). A large number of *WRKY* DNA-binding proteins are implicated in the transcriptional activation of defense related genes in response to pathogens (Ryu et al., 2006; Eulgem and Somssich, 2007). A systematic expression analysis of *WRKY*s in rice revealed that the expression of 15 out of 45 *WRKY* genes significantly increased in an incompatible rice-pathogen interaction (Ryu et al., 2006). The expression of two and three *WRKY* genes increased in response to SA- and JA- treatments, respectively. *CAZFP1*, a C2H2-type zinc-finger transcription factor, functions as a pathogen-induced early-defense gene to enhance disease resistance (Kim et al., 2004). Overexpression of the *CAZFP1* in stably transformed *Arabidopsis* plants enhanced the resistance against infection by *Pseudomonas syringae* pv. *tomato*. A novel CCCH-type zinc finger protein GhZFP1 from cotton also enhances fungal disease resistance in transgenic tobacco by interacting with two other proteins (Guo et al., 2009). The expression of *ERF* and *NAC* genes is regulated by plant hormones, such as JA, SA and ethylene, and several lines of research have confirmed that they participate in the regulation of disease resistance pathways (Gutterson and Reuber, 2004; He et al., 2005; Xia et al., 2010a,b; Nuruzzaman et al., 2012, 2013; Zheng et al., 2012). In rice seedlings, 19 *NAC* genes displayed higher expression levels after PSV infection, and 13 *NAC* genes upon RTSV infection (Nuruzzaman et al., 2010). Several NAC proteins can act as repressors as well as activators in virus replication by directly interacting with virus-encoded proteins (Xie et al., 1999; Ren et al., 2000; Selth et al., 2005). The above findings imply that different types of transcription factors have developed a novel significant way in regulating the expression of a large set of immune-related genes, which are activated by defense responses to PRSV in transgenic papaya after years of plant-pathogen interactions. Numerous DEGs were also predicted to be hormone-related genes in this study. This finding corroborates earlier findings, which showed that the phytohormones ethylene, SA and JA play a central role in the regulation of plant immune responses (Vlot et al., 2009; Robert-Seilaniantz et al., 2011). Suppression of the JA signaling component *COI1* ortholog affected tobacco *N* resistance (Liu et al., 2004). Other plant hormones that have been thoroughly described to function in the regulation of plant growth, development and the response to abiotic stresses, such as auxins, ABA, brassinosteroid, gibberellin, and cytokinin, have recently emerged as crucial regulators of plant immunity (Mauch-Mani and Mauch, 2005; Kazan and Manners, 2009; Ton et al., 2009; Wang and Fu, 2011). All these lines of evidence might suggest that hormonal signaling involving crosstalk between ABA, SA, ethylene, auxin and other hormones may be essential in the papaya pathogen response.

Transport proteins, embedded within plasma membrane, cytoplasm or nucleus, function in both active and passive transport to regulate molecules moving in and out of cell. Some ABC transporters are known to play a role in resistance to pathogens (Krattinger et al., 2009; Wang et al., 2013a; Goyer et al., 2015). In barley, ABC transporters are highly expressed upon inoculation with barley yellow dwarf virus (Wang et al., 2013a). The down-regulation of ABC transporters in the papaya transgenic cultivar SunUp might suggest that fewer ABC transporters were implicated in transgenic papaya since its divergence from non-transgenic papaya Sunset due to the inherent resistance of SunUp. Many nitrate and zinc transporters were highly expressed in SunUp compared to that in Sunset. Nitrogen is the mineral nutrient needed in greatest abundance by plants because it is a major component of amino acids, nucleic acids, ATP (adenosine triphosphate) and chlorophyII (Crawford, 1995). Zinc is needed in small but if its amount is not adequate, plants will suffer from physiological stress brought about by the dysfunction of several enzyme systems and other metabolic functions in which zinc plays a vital part (Alloway, 2004). Both nitrate and zinc are essential for the normal healthy growth of plants. Therefore, the better growth of SunUp plants (Figure S1A) could be easily explained by the up-regulation of nitrate and zinc transporters. Notably, two genes, homologous to the *Arabidopsis thaliana* zinc transporter 5 precursor and nodulin MtN21, were found to be exclusively expressed in SunUp, suggesting these transgenic plant lines may have induced new transporters for environmental adaptation. The role of nodulin MtN21-like genes is not yet known. The maize nodulin MtN21-like gene is probably involved in transport of a component related to vascular tissue assembly (Guillaumie et al., 2008).

### Increased rate of as in the PRSV CP transgene in stress response

Alternative splicing is a regulated process during gene expression that results in the production of multiple mRNAs from a single gene by variable selection of splice sites in the precursor-mRNA. RNA-Seq is a proven high-throughput and cost-saving technology that identifies AS events. In our analysis, we focused on six types of AS events: SE, RI, A5SS, A3SS, 5UTR, and 3UTR, among which, SE was the most abundant type, accounting for more than 50% of total AS events. A greater number of AS events were found in SunUp compared to Sunset (68,455 vs. 67,686), while the number of genes undergoing AS in both cultivars was similar. These findings suggest that ~39% of papaya genes potentially undergo the AS process, but AS events occurring in papaya increased upon PRSV CP transgene insertion. This observed percentage of AS events in papaya (39%) is comparable to that observed in *A. thaliana* (42%) and much higher than rice (33%) (Wang and Brendel, 2006; Simpson et al., 2008; Syed et al., 2012). 610 genes in Sunset and 611 genes in SunUp were exclusively alternatively spliced (Figure 7A), indicating that transgenic papaya diverged from its progenitor to undergo exclusive alternatively splicing events in responses to biotic and abiotic stresses. The biological role of AS in photosynthesis, defense responses and grain quality in rice have been verified (Reddy, 2007). More than 60% of intron-containing genes undergo alternative splicing in plants (Syed et al., 2012). Nevertheless, little is known of the alternative splicing of papaya genes at the genome-wide level. Despite having a significantly larger genome than *Arabidopsis thaliana*, it is estimated that papaya has fewer genes than other sequenced genomes of flowering plants (Ming et al., 2008, 2012). This is partly due to the absence of further genome wide duplication detected in papaya since the ancient triplication event shared by eudicots. The small set of genes in papaya implies that papaya may have a relatively higher frequency of AS events to synthesize various types of proteins to maintain basic functions during development and in response to environmental signals compared to other plants. A nonlinear correlation between NBS gene number and total gene number revealed that papaya has significantly fewer NBS genes (~0.2% of total genes), which could explain the susceptibility of papaya to multiple pathogens (Nishijima, 2002; Porter et al., 2009). Intriguingly, numerous splice variants were identified in the papaya NBS gene family, suggesting that alternative splicing may play a vital role in contributing to the diversity of NBS-encoding genes.

## Conclusions

This study is the first large scale transcriptome RNA-Seq analysis comparing a PRSV resistant transgenic papaya SunUp and its progenitor cultivar Sunset. Genome-wide transcriptional profiling and analysis of alternative splicing events in the present work indicated that most biological processes remain shared by the two cultivars SunUp and Sunset. Numerous DEGs were identified, of which up-regulated genes were mainly related to stress tolerance and pathogen resistance, including various TFs, TPs as well as genes involved in hormone signaling pathways. These findings demonstrated that transgenic papaya has the potential to improve the abiotic and biotic tolerance in addition to PRSV resistance. The differential expressions of candidate DEGs inferred from RNA-Seq were confirmed by qRT-PCR, demonstrating the accuracy and reliability of the RNA-Seq data. GO and KEGG enrichment analysis established that two cultivars trigger a different cascade of molecular changes, deepening the understanding of the complex molecular and cellular events during various biological pathways in these two cultivars. Further investigations will be needed to elucidate the specific mechanisms of genes associated with TFs, TPs, and HRGs and of genes belonging to GO terms/KEGG pathways significantly enriched. These genes involved in pathways of interest may be important targets for biotechnological manipulation to improve papaya stress tolerance and disease resistance, particularly in the early development stages.

## Deposited data

The Illumina RNA-sequencing raw reads of PRSV susceptible cultivar Sunset and PRSV resistant transgenic papaya cultivar SunUp are available from the NCBI Sequence Read Archive database (SRA; http://www.ncbi.nlm.nih.gov/sra/) under project Accession number of SRP075196.

## Author contributions

RM and RC conceived the study and designed the experiments. JF, AL, WQ, HC, and MU carried out the experiments and analyzed the data. JF and RM wrote the manuscript. All authors read and approved the final paper.

### Conflict of interest statement

The authors declare that the research was conducted in the absence of any commercial or financial relationships that could be construed as a potential conflict of interest.
