# Supplementary material for: Transcriptome Profiling Revealed Stress-Induced and Disease Resistance Genes Up-Regulated in PRSV Resistant Transgenic Papaya
Source: Front Plant Sci. 2016 Jun 16;7:855. doi: 10.3389/fpls.2016.00855 (PMC4909764; doi:10.3389/fpls.2016.00855)
Supplement: Supplementary file 1 [file DataSheet1.doc]

**
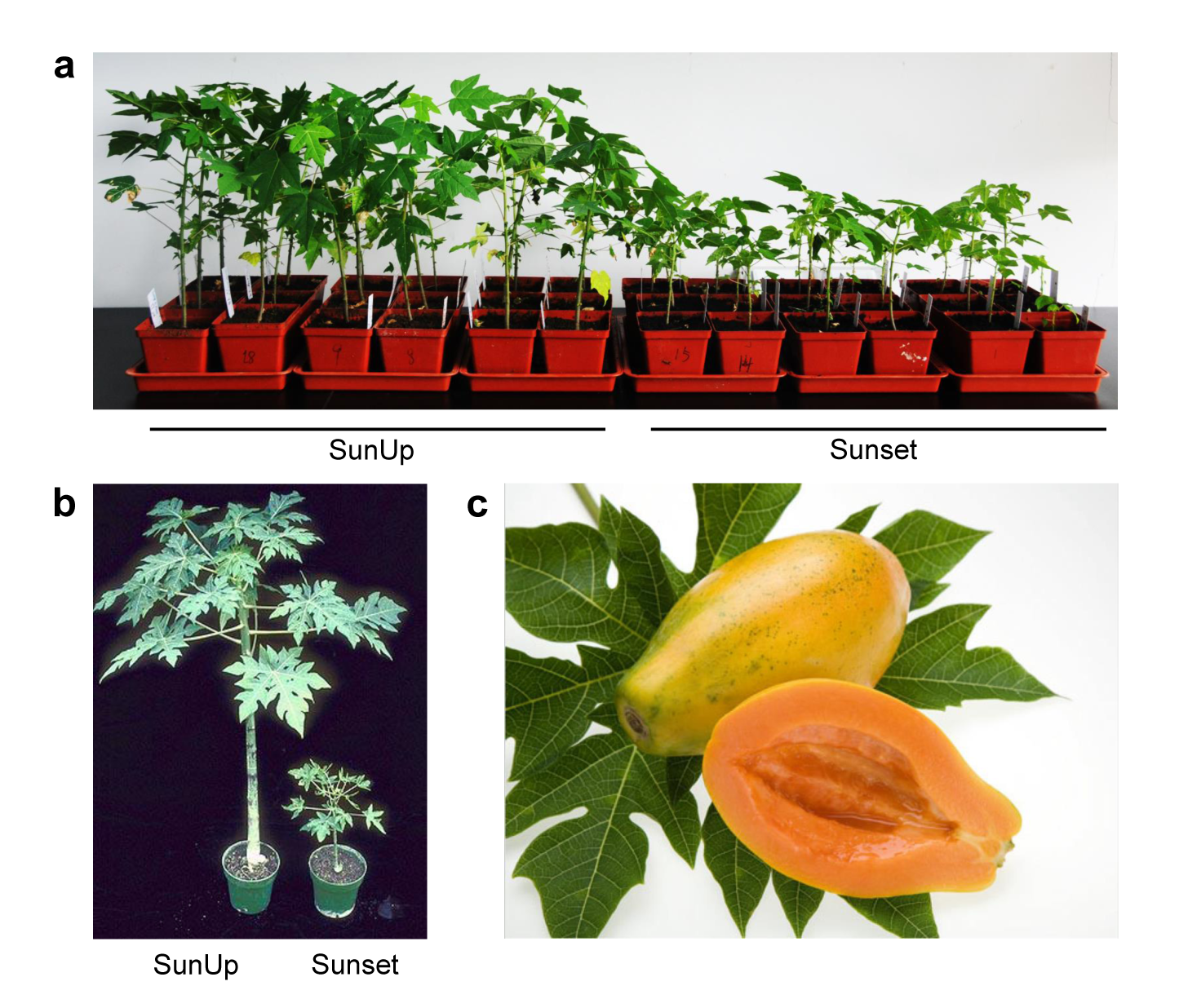
**

**Fig. S1 Agronomic trait performance of PRSV resistant transgenic papaya SunUp and PRSV susceptible papaya Sunset. a.** Seedlings of transgenic papaya SunUp grow more vigorously than non-transgenic papaya Sunset under the same conditions prior to infection with PRSV; **b.** Transgenic papaya SunUp showing resistance to PRSV HA compared to infected non-transgenic papaya Sunset. (from Gonsalves, 2004, *AgBi*o*Forum, 7*, p. 37); **c.** SunUp and Sunset share pink-tinged flesh with a melting texture. (from Ming, 1998, p. 102).


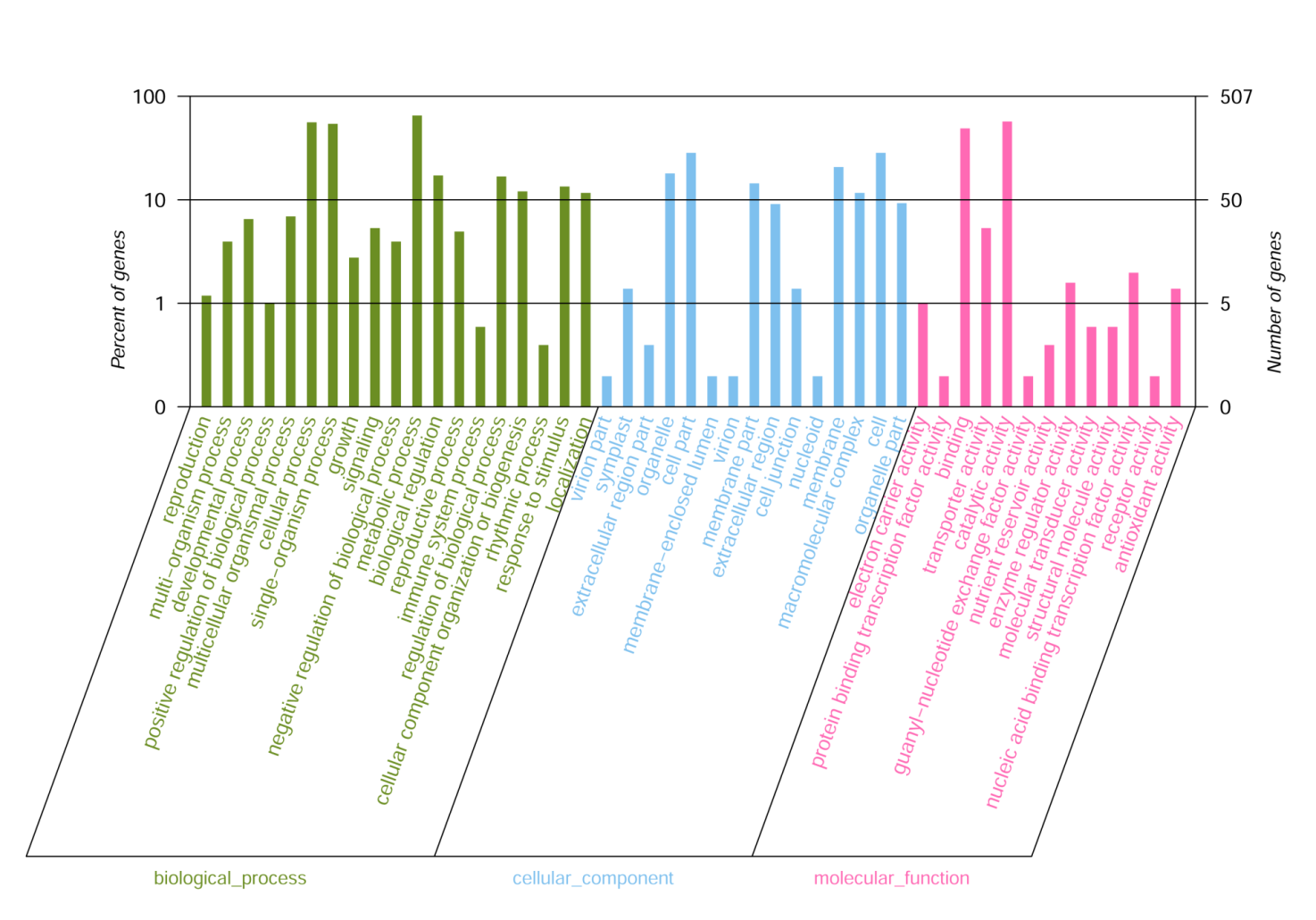


**Fig. S2 Histogram of level 2 Gene Ontology classification of all DEGs compared between the SunUp and Sunset transcriptome.** The results are summarized in three main categories: biological process, cellular component and molecular function. The right *Y*-axis indicates the number of genes in a category. The left *Y*-axis indicates the percentage of a specific category of genes in that main category.

**
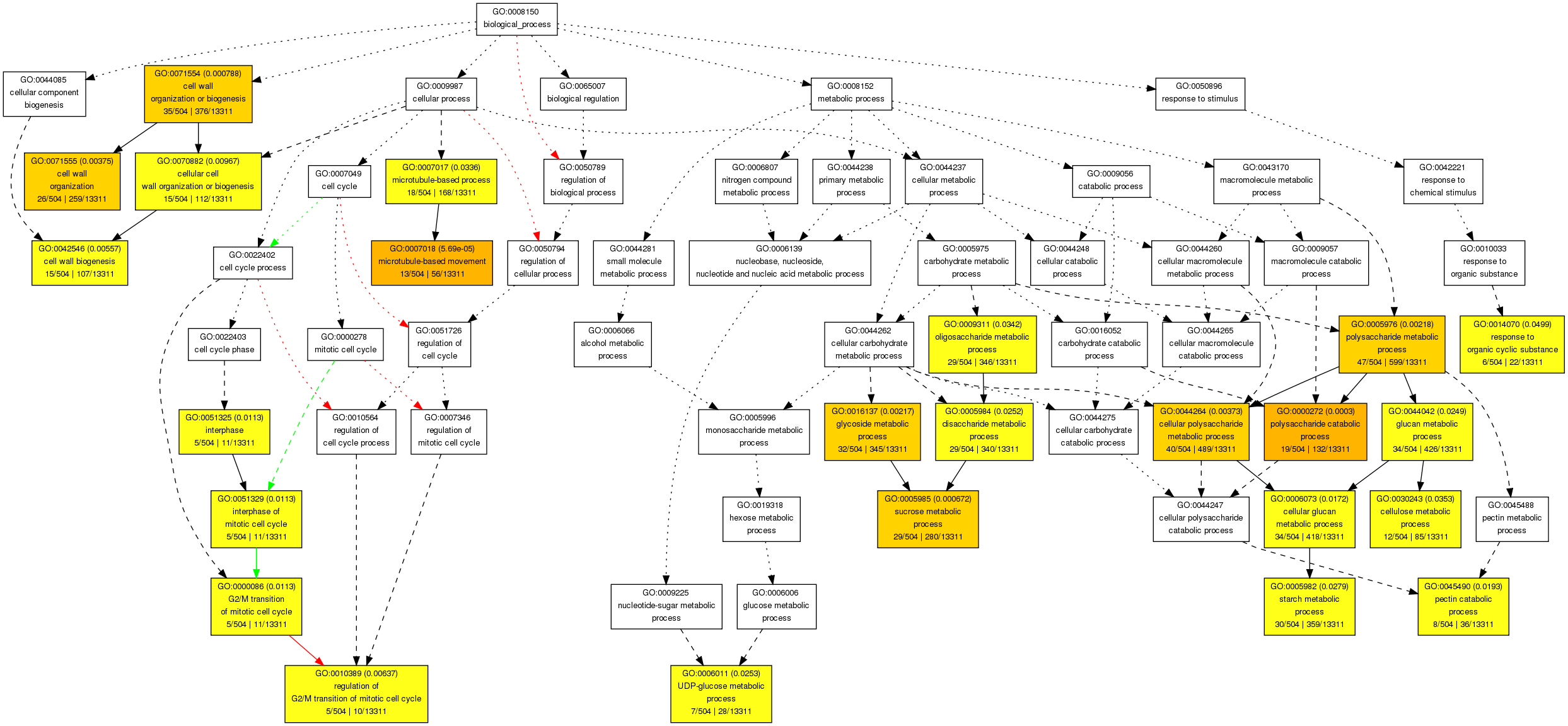
**

**Fig. S3 AgriGO results of DEGs in SU-NP and SS-NP based on SEA analysis.** Significance level of enrichment is displayed by color scale. White indicates no significant enrichment; color transitions from yellow to red to indicate strength of significance. Ratios at the bottom of each GO box represent the number of genes in the input list matching that GO term vs. the number of total genes in the input list, total genes in the background genome set matching that GO term vs. total genes in the background set. The adjusted *p*-value for each enriched GO term is indicated in the parentheses ( ) at the top of each colored box.


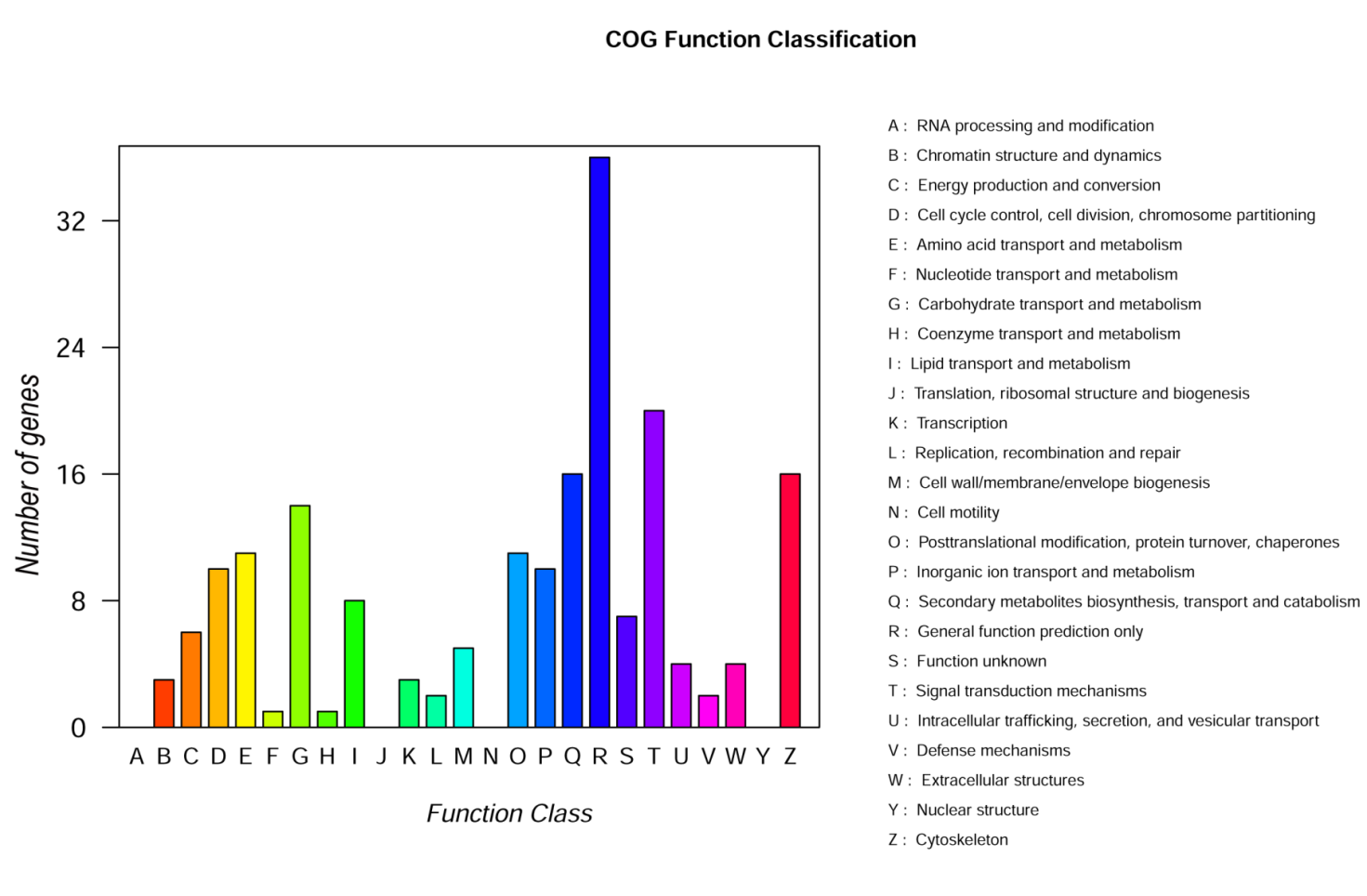


**Fig. S4 Histogram of KOG classification of DEGs compared between SunUp and Sunset transcriptomes.** All detected DEGs were blasted to STRING 9.0 for further annotation based on Cluster of Orthologous Groups (COG) of protein categories. Capital letters on the *X*-axis indicate 25 categories of KOG. The *Y*-axis indicates the percentage of the number of genes.

**Table S1a.** Level 2 Gene Ontology (GO) annotation of 475 up-regulated DEGs in transgenic papaya SunUp compared to the donor variety Sunset into three main ontologies: **a** Molecular function. **b** Biological process and **c** Cellular component.

Total gene number with GO ID: 268

| **Term type** | **Function description** | **No.** | **Percentage** | **GO** |
| --- | --- | --- | --- | --- |
| **Molecular function** | electron carrier activity | 5 | 1.87% | GO:0009055 |
|  | protein binding transcription factor activity | 1 | 0.37% | GO:0000988 |
|  | binding | 123 | 45.90% | GO:0005488 |
|  | transporter activity | 23 | 8.58% | GO:0005215 |
|  | catalytic activity | 154 | 57.46% | GO:0003824 |
|  | guanyl-nucleotide exchange factor activity | 0 | 0.00% |  |
|  | nutrient reservoir activity | 1 | 0.37% | GO:0045735 |
|  | enzyme regulator activity | 4 | 1.49% | GO:0030234 |
|  | structural molecule activity | 0 | 0.00% |  |
|  | molecular transducer activity | 3 | 1.12% | GO:0060089 |
|  | receptor activity | 1 | 0.37% | GO:0004872 |
|  | nucleic acid binding transcription factor activity | 7 | 2.61% | GO:0001071 |
|  | antioxidant activity | 2 | 0.75% | GO:0016209 |
| **Cellular component** | virion part | 1 | 0.37% | GO:0044423 |
|  | symplast | 1 | 0.37% | GO:0055044 |
|  | extracellular region part | 2 | 0.75% | GO:0044421 |
|  | organelle | 28 | 10.45% | GO:0043226 |
|  | cell part | 47 | 17.54% | GO:0044464 |
|  | membrane-enclosed lumen | 1 | 0.37% | GO:0031974 |
|  | virion | 1 | 0.37% | GO:0019012 |
|  | membrane part | 35 | 13.06% | GO:0044425 |
|  | extracellular region | 16 | 5.97% | GO:0005576 |
|  | cell junction | 1 | 0.37% | GO:0030054 |
|  | nucleoid | 1 | 0.37% | GO:0009295 |
|  | membrane | 54 | 20.15% | GO:0016020 |
|  | macromolecular complex | 20 | 7.46% | GO:0032991 |
|  | cell | 47 | 17.54% | GO:0005623 |
|  | organelle part | 11 | 4.10% | GO:0044422 |
| **Bological process** | reproduction | 2 | 0.75% | GO:0000003 |
|  | multi-organism process | 12 | 4.48% | GO:0051704 |
|  | developmental process | 11 | 4.10% | GO:0032502 |
|  | positive regulation of biological process | 3 | 1.12% | GO:0048518 |
|  | multicellular organismal process | 14 | 5.22% | GO:0032501 |
|  | cellular process | 152 | 56.72% | GO:0009987 |
|  | single-organism process | 142 | 52.99% | GO:0044699 |
|  | growth | 4 | 1.49% | GO:0040007 |
|  | signaling | 21 | 7.84% | GO:0023052 |
|  | negative regulation of biological process | 7 | 2.61% | GO:0048519 |
|  | metabolic process | 171 | 63.81% | GO:0008152 |
|  | biological regulation | 42 | 15.67% | GO:0065007 |
|  | reproductive process | 10 | 3.73% | GO:0022414 |
|  | immune system process | 3 | 1.12% | GO:0002376 |
|  | regulation of biological process | 42 | 15.67% | GO:0050789 |
|  | cellular component organization or biogenesis | 14 | 5.22% | GO:0071840 |
|  | rhythmic process | 1 | 0.37% | GO:0048511 |
|  | response to stimulus | 43 | 16.04% | GO:0050896 |
|  | localization | 45 | 16.79% | GO:0051179 |

**Table S1b.** Level 2 Gene Ontology (GO) annotation of 367 down-regulated DEGs in transgenic papaya SunUp compared with donor variety Sunset grouped in three main ontologies: **a** Molecular function. **b** Biological process and **c** Cellular component.

Total gene number with GO ID: 239

| **Term type** | **Function description** | **No.** | **Percentage** | **GO** |
| --- | --- | --- | --- | --- |
| **Molecular function** | electron carrier activity | 0 | 0.00% |  |
|  | protein binding transcription factor activity | 0 | 0.00% |  |
|  | binding | 125 | 52.30% | GO:0005488 |
|  | transporter activity | 4 | 1.67% | GO:0005215 |
|  | catalytic activity | 135 | 56.49% | GO:0003824 |
|  | guanyl-nucleotide exchange factor activity | 1 | 0.42% | GO:0005085 |
|  | nutrient reservoir activity | 1 | 0.42% | GO:0045735 |
|  | enzyme regulator activity | 4 | 1.67% | GO:0030234 |
|  | structural molecule activity | 3 | 1.26% | GO:0005198 |
|  | molecular transducer activity | 0 | 0.00% |  |
|  | receptor activity | 0 | 0.00% |  |
|  | nucleic acid binding transcription factor activity | 3 | 1.26% | GO:0001071 |
|  | antioxidant activity | 5 | 2.09% | GO:0016209 |
| **Cellular component** | virion part | 0 | 0.00% |  |
|  | symplast | 6 | 2.51% | GO:0055044 |
|  | extracellular region part | 0 | 0.00% |  |
|  | organelle | 63 | 26.36% | GO:0043226 |
|  | cell part | 97 | 40.59% | GO:0044464 |
|  | membrane-enclosed lumen | 0 | 0.00% |  |
|  | virion | 0 | 0.00% |  |
|  | membrane part | 38 | 15.90% | GO:0044425 |
|  | extracellular region | 30 | 12.55% | GO:0005576 |
|  | cell junction | 6 | 2.51% | GO:0030054 |
|  | nucleoid | 0 | 0.00% |  |
|  | membrane | 51 | 21.34% | GO:0016020 |
|  | macromolecular complex | 39 | 16.32% | GO:0032991 |
|  | cell | 97 | 40.59% | GO:0005623 |
|  | organelle part | 36 | 15.06% | GO:0044422 |
| **Bological process** | reproduction | 4 | 1.67% | GO:0000003 |
|  | multi-organism process | 8 | 3.35% | GO:0051704 |
|  | developmental process | 22 | 9.21% | GO:0032502 |
|  | positive regulation of biological process | 2 | 0.84% | GO:0048518 |
|  | multicellular organismal process | 21 | 8.79% | GO:0032501 |
|  | cellular process | 132 | 55.23% | GO:0009987 |
|  | single-organism process | 132 | 55.23% | GO:0044699 |
|  | growth | 10 | 4.18% | GO:0040007 |
|  | signaling | 6 | 2.51% | GO:0023052 |
|  | negative regulation of biological process | 13 | 5.44% | GO:0048519 |
|  | metabolic process | 159 | 66.53% | GO:0008152 |
|  | biological regulation | 45 | 18.83% | GO:0065007 |
|  | reproductive process | 15 | 6.28% | GO:0022414 |
|  | immune system process | 0 | 0.00% |  |
|  | regulation of biological process | 43 | 17.99% | GO:0050789 |
|  | cellular component organization or biogenesis | 47 | 19.67% | GO:0071840 |
|  | rhythmic process | 1 | 0.42% | GO:0048511 |
|  | response to stimulus | 25 | 10.46% | GO:0050896 |
|  | localization | 14 | 5.86% | GO:0051179 |

**Table S2.** GO enrichment analyses for the DEGs.

| **GO term** | **Ontology** | **Description** | **Number in input list** | **Number in BG/Ref** | ***p*-value** | **FDR** | **-log_10_ FDR** |
| --- | --- | --- | --- | --- | --- | --- | --- |
| GO:0007018 | P | microtubule-based movement | 13 | 56 | 1.50E-07 | 5.70E-05 | 4.24412514 |
| GO:0000272 | P | polysaccharide catabolic process | 19 | 132 | 8.10E-07 | 0.0003 | 3.52287875 |
| GO:0005985 | P | sucrose metabolic process | 29 | 280 | 1.80E-06 | 0.00067 | 3.1739252 |
| GO:0071554 | P | cell wall organization or biogenesis | 35 | 376 | 2.10E-06 | 0.00079 | 3.10237291 |
| GO:0016137 | P | glycoside metabolic process | 32 | 345 | 5.90E-06 | 0.0022 | 2.65757732 |
| GO:0005976 | P | polysaccharide metabolic process | 47 | 599 | 5.90E-06 | 0.0022 | 2.65757732 |
| GO:0044264 | P | cellular polysaccharide metabolic process | 40 | 489 | 1.00E-05 | 0.0037 | 2.43179828 |
| GO:0071555 | P | cell wall organization | 26 | 259 | 1.00E-05 | 0.0038 | 2.4202164 |
| GO:0042546 | P | cell wall biogenesis | 15 | 107 | 1.50E-05 | 0.0056 | 2.25181197 |
| GO:0010389 | P | regulation of G2/M transition of mitotic cell cycle | 5 | 10 | 1.70E-05 | 0.0064 | 2.19382003 |
| GO:0070882 | P | cellular cell wall organization or biogenesis | 15 | 112 | 2.60E-05 | 0.0097 | 2.01322827 |
| GO:0000086 | P | G2/M transition of mitotic cell cycle | 5 | 11 | 3.00E-05 | 0.011 | 1.95860731 |
| GO:0051329 | P | interphase of mitotic cell cycle | 5 | 11 | 3.00E-05 | 0.011 | 1.95860731 |
| GO:0051325 | P | interphase | 5 | 11 | 3.00E-05 | 0.011 | 1.95860731 |
| GO:0006073 | P | cellular glucan metabolic process | 34 | 418 | 4.60E-05 | 0.017 | 1.76955108 |
| GO:0045490 | P | pectin catabolic process | 8 | 36 | 5.20E-05 | 0.019 | 1.7212464 |
| GO:0044042 | P | glucan metabolic process | 34 | 426 | 6.70E-05 | 0.025 | 1.60205999 |
| GO:0005984 | P | disaccharide metabolic process | 29 | 340 | 6.80E-05 | 0.025 | 1.60205999 |
| GO:0006011 | P | UDP-glucose metabolic process | 7 | 28 | 6.80E-05 | 0.025 | 1.60205999 |
| GO:0005982 | P | starch metabolic process | 30 | 359 | 7.50E-05 | 0.028 | 1.55284197 |
| GO:0007017 | P | microtubule-based process | 18 | 168 | 9.00E-05 | 0.034 | 1.46852108 |
| GO:0009311 | P | oligosaccharide metabolic process | 29 | 346 | 9.20E-05 | 0.034 | 1.46852108 |
| GO:0030243 | P | cellulose metabolic process | 12 | 85 | 9.50E-05 | 0.035 | 1.45593196 |
| GO:0014070 | P | response to organic cyclic substance | 6 | 22 | 0.00013 | 0.05 | 1.30103 |
| GO:0003777 | F | microtubule motor activity | 13 | 55 | 1.20E-07 | 1.40E-05 | 4.85387196 |
| GO:0008017 | F | microtubule binding | 14 | 76 | 1.10E-06 | 0.00012 | 3.92081875 |
| GO:0015631 | F | tubulin binding | 14 | 78 | 1.50E-06 | 0.00017 | 3.76955108 |
| GO:0003774 | F | motor activity | 13 | 71 | 2.70E-06 | 0.00031 | 3.50863831 |
| GO:0008092 | F | cytoskeletal protein binding | 17 | 121 | 4.10E-06 | 0.00047 | 3.32790214 |
| GO:0045330 | F | aspartyl esterase activity | 8 | 32 | 2.10E-05 | 0.0024 | 2.61978876 |
| GO:0030599 | F | pectinesterase activity | 9 | 43 | 3.00E-05 | 0.0034 | 2.46852108 |
| GO:0016760 | F | cellulose synthase (UDP-forming) activity | 7 | 25 | 3.10E-05 | 0.0035 | 2.45593196 |
| GO:0016759 | F | cellulose synthase activity | 7 | 26 | 4.10E-05 | 0.0047 | 2.32790214 |
| GO:0004857 | F | enzyme inhibitor activity | 8 | 47 | 0.00037 | 0.043 | 1.36653154 |
| GO:0005576 | C | extracellular region | 46 | 379 | 1.70E-11 | 1.10E-09 | 8.95860731 |
| GO:0005618 | C | cell wall | 26 | 168 | 2.00E-09 | 1.20E-07 | 6.92081875 |
| GO:0030312 | C | external encapsulating structure | 26 | 170 | 2.60E-09 | 1.60E-07 | 6.79588002 |
| GO:0015630 | C | microtubule cytoskeleton | 22 | 141 | 2.70E-08 | 1.70E-06 | 5.76955108 |
| GO:0005871 | C | kinesin complex | 13 | 55 | 1.20E-07 | 7.40E-06 | 5.13076828 |
| GO:0005856 | C | cytoskeleton | 26 | 224 | 7.10E-07 | 4.30E-05 | 4.36653154 |
| GO:0044430 | C | cytoskeletal part | 23 | 191 | 1.50E-06 | 9.40E-05 | 4.02687215 |
| GO:0048046 | C | apoplast | 16 | 101 | 1.60E-06 | 9.60E-05 | 4.01772877 |
| GO:0005875 | C | microtubule associated complex | 13 | 68 | 1.60E-06 | 0.0001 | 4 |
| GO:0045298 | C | tubulin complex | 14 | 80 | 2.00E-06 | 0.00012 | 3.92081875 |
| GO:0005874 | C | microtubule | 15 | 93 | 2.60E-06 | 0.00016 | 3.79588002 |

| **Table S3.** Statistical enrichment analysis for KEGG pathways.  ##Databases: KEGG PATHWAY  ##Statistical test method: hypergeometric test / Fisher's exact test  ##FDR correction method: Benjamini and Hochberg | | |  |  |  |  |
| --- | --- | --- | --- | --- | --- | --- |
| **Term** | **ID** | **Gene number** | **Background number** | ***p*-Value** | **FDR** | **Rich Factor** |
| Phenylpropanoid biosynthesis | ko00940 | 11 | 86 | 0.000156194 | 0.01952423 | 0.127906977 |
| Cutin, suberine and wax biosynthesis | ko00073 | 4 | 10 | 0.000644591 | 0.038809135 | 0.4 |
| Starch and sucrose metabolism | ko00500 | 11 | 115 | 0.001475039 | 0.038809135 | 0.095652174 |
| Chemical carcinogenesis | ko05204 | 6 | 36 | 0.001519621 | 0.038809135 | 0.166666667 |
| Pentose and glucuronate interconversions | ko00040 | 7 | 50 | 0.001552365 | 0.038809135 | 0.14 |
| Drug metabolism - cytochrome P450 | ko00982 | 6 | 38 | 0.001944357 | 0.039065888 | 0.157894737 |
| Metabolism of xenobiotics by cytochrome P450 | ko00980 | 6 | 39 | 0.00218769 | 0.039065888 | 0.153846154 |

**Table S4.** The 118 differentially expressed genes encoding transcription factors (TFs) between two cultivars.

| **Gene ID** | ***Arabidopsis*** | **Gene annotation** | **Expression level (FPKM)** | | **Cluster** |
| --- | --- | --- | --- | --- | --- |
|  |  |  | **SS-NP** | **SU-NP** |  |
| evm.TU.supercontig_58.37 | AT5G43650.1 | Basic helix-loop-helix (bHLH) DNA-binding superfamily protein | 6.27135 | 223.272 | K1 |
| evm.TU.supercontig_80.96 | AT3G15510.1 | NAC domain containing protein 2 | 1.01731 | 25.93 | K1 |
| evm.TU.supercontig_807.3 | AT5G13080.1 | WRKY DNA-binding protein 75 | 2.86936 | 63.0173 | K1 |
| evm.TU.supercontig_50.27 | AT3G23230.1 | Integrase-type DNA-binding superfamily protein | 3.81205 | 79.7241 | K1 |
| evm.TU.supercontig_26.302 | AT4G12020.2 | Protein kinase family protein | 2.3915 | 77.3382 | K1 |
| evm.TU.supercontig_190.29 | AT2G28710.1 | C2H2-type zinc finger family protein | 4.24345 | 109.473 | K1 |
| evm.TU.supercontig_34.60 | AT1G34670.1 | Myb domain protein 93 | 26.4289 | 555.113 | K1 |
| evm.TU.supercontig_126.44 | AT5G26170.1 | WRKY DNA-binding protein 50 | 3.85459 | 99.2079 | K1 |
| evm.TU.supercontig_7.130 | AT3G20840.1 | Integrase-type DNA-binding superfamily protein | 0.395171 | 1.77566 | K2 |
| evm.TU.supercontig_66.100 | AT4G12020.2 | Protein kinase family protein | 0.469227 | 1.9332 | K2 |
| evm.TU.supercontig_544.1 | AT4G12020.2 | Protein kinase family protein | 0.454824 | 2.46352 | K2 |
| evm.TU.supercontig_55.62 | AT1G72210.1 | Basic helix-loop-helix (bHLH) DNA-binding superfamily protein | 0 | 2.13653 | K2 |
| evm.TU.supercontig_519.1 | AT4G25440.1 | Zinc finger WD40 repeat protein 1 | 1.39697 | 6.5147 | K2 |
| evm.TU.supercontig_66.70 | AT4G12020.2 | Protein kinase family protein | 1.47264 | 6.17666 | K2 |
| evm.TU.supercontig_5.28 | AT2G36270.1 | Basic-leucine zipper (bZIP) transcription factor family protein | 0.696095 | 3.65288 | K2 |
| evm.TU.supercontig_74.83 | AT4G25480.1 | Dehydration response element B1A | 11.6146 | 75.5762 | K2 |
| evm.TU.supercontig_34.213 | AT4G12020.2 | Protein kinase family protein | 21.0464 | 131.526 | K2 |
| evm.TU.supercontig_152.35 | AT1G62300.1 | WRKY family transcription factor | 22.0205 | 136.961 | K2 |
| evm.TU.supercontig_9.35 | AT5G49520.1 | WRKY DNA-binding protein 48 | 17.2988 | 106.025 | K2 |
| evm.TU.supercontig_14.99 | AT5G61430.1 | NAC domain containing protein 100 | 6.85613 | 43.7501 | K2 |
| evm.TU.supercontig_21.63 | AT1G71520.1 | Integrase-type DNA-binding superfamily protein | 9.59186 | 59.684 | K2 |
| evm.TU.supercontig_3.487 | AT4G12020.2 | Protein kinase family protein | 2.49472 | 19.0917 | K2 |
| evm.TU.supercontig_129.23 | AT1G30330.2 | Auxin response factor 6 | 49.3207 | 288.859 | K2 |
| evm.TU.supercontig_195.12 | AT3G44350.2 | NAC domain containing protein 61 | 28.5282 | 161.284 | K2 |
| evm.TU.supercontig_106.59 | AT5G67450.1 | Zinc finger protein 1 | 77.3986 | 433.125 | K2 |
| evm.TU.supercontig_95.50 | AT4G12020.3 | Protein kinase family protein | 16.5506 | 126.303 | K2 |
| evm.TU.supercontig_3.494 | AT1G68320.1 | Myb domain protein 62 | 23.1542 | 170.373 | K2 |
| evm.TU.supercontig_3.54 | AT4G23810.1 | WRKY family transcription factor | 67.3238 | 442.734 | K2 |
| evm.TU.supercontig_27.16 | AT4G12020.1 | Protein kinase family protein | 4.49573 | 34.731 | K2 |
| evm.TU.supercontig_20.220 | AT4G12020.3 | Protein kinase family protein | 18.0854 | 120.054 | K2 |
| evm.TU.supercontig_12.9 | AT4G12020.2 | Protein kinase family protein | 3.27164 | 26.05 | K2 |
| evm.TU.supercontig_19.263 | AT4G12020.2 | Protein kinase family protein | 44.0379 | 307.074 | K2 |
| evm.TU.supercontig_594.1 | AT5G18270.1 | NAC domain containing protein 87 | 7.7607 | 57.9356 | K2 |
| evm.TU.supercontig_104.8 | AT5G06510.2 | Nuclear factor Y, subunit A10 | 2.45138 | 22.29 | K2 |
| evm.TU.supercontig_61.12 | AT5G56960.1 | Basic helix-loop-helix (bHLH) DNA-binding family protein | 1.351 | 7.10693 | K2 |
| evm.TU.supercontig_28.106 | AT4G12020.3 | Protein kinase family protein | 1.87212 | 8.65488 | K2 |
| evm.TU.supercontig_90.41 | AT4G17980.1 | NAC domain containing protein 71 | 3.13455 | 13.6563 | K2 |
| evm.TU.supercontig_8.114 | AT1G64100.2 | Pentatricopeptide (PPR) repeat-containing protein | 3.42122 | 14.8948 | K2 |
| evm.TU.supercontig_19.214 | AT5G05790.1 | Duplicated homeodomain-like superfamily protein | 0.910206 | 6.07338 | K2 |
| evm.TU.supercontig_3.486 | AT4G12020.2 | Protein kinase family protein | 14.7707 | 61.3578 | K2 |
| evm.TU.supercontig_2.76 | AT1G14600.1 | Homeodomain-like superfamily protein | 17.9958 | 73.8746 | K2 |
| evm.TU.supercontig_55.149 | AT4G36990.1 | Heat shock factor 4 | 227.95 | 929.6 | K2 |
| evm.TU.supercontig_65.57 | AT4G12020.3 | Protein kinase family protein | 3.07363 | 15.4992 | K2 |
| evm.TU.supercontig_50.20 | AT2G31180.1 | Myb domain protein 14 | 109.381 | 445.607 | K2 |
| evm.TU.supercontig_37.27 | AT1G01720.1 | NAC (No Apical Meristem) domain transcriptional regulator superfamily protein | 143.789 | 576.224 | K2 |
| evm.TU.supercontig_57.20 | AT4G01720.1 | WRKY family transcription factor | 8.62852 | 37.7123 | K2 |
| evm.TU.supercontig_20.69 | AT5G51990.1 | C-repeat-binding factor 4 | 43.2345 | 177.084 | K2 |
| evm.TU.supercontig_64.137 | AT4G12020.2 | Protein kinase family protein | 0.828546 | 6.33204 | K2 |
| evm.TU.supercontig_129.37 | AT4G37850.1 | Basic helix-loop-helix (bHLH) DNA-binding superfamily protein | 18.6208 | 77.6709 | K2 |
| evm.TU.supercontig_177.21 | AT4G12020.2 | Protein kinase family protein | 14.2121 | 62.7309 | K2 |
| evm.TU.supercontig_163.28 | AT2G37430.1 | C2H2 and C2HC zinc fingers superfamily protein | 4.04717 | 19.8343 | K2 |
| evm.TU.supercontig_919.2 | AT5G64810.1 | WRKY DNA-binding protein 51 | 12.8434 | 55.7551 | K2 |
| evm.TU.supercontig_3.77 | AT5G57660.1 | CONSTANS-like 5 | 39.8852 | 166.965 | K2 |
| evm.TU.supercontig_111.23 | AT5G61430.1 | NAC domain containing protein 100 | 8.31575 | 37.3013 | K2 |
| evm.TU.supercontig_81.18 | AT4G12020.2 | Protein kinase family protein | 27.6504 | 122.03 | K2 |
| evm.TU.supercontig_14.83 | AT3G57480.1 | Zinc finger (C2H2 type, AN1-like) family protein | 26.1943 | 115.174 | K2 |
| evm.TU.supercontig_104.90 | AT5G22380.1 | NAC domain containing protein 90 | 24.0289 | 107.401 | K2 |
| evm.TU.supercontig_1.401 | AT4G12020.3 | Protein kinase family protein | 64.7158 | 285.065 | K2 |
| evm.TU.supercontig_83.80 | AT5G44210.1 | ERF domain protein 9 | 70.8987 | 323.625 | K2 |
| evm.TU.supercontig_6.74 | AT5G25160.1 | Zinc finger protein 3 | 0.831213 | 7.33186 | K2 |
| evm.TU.supercontig_190.34 | AT3G46130.1 | Myb domain protein 48 | 1.53937 | 10.5484 | K2 |
| evm.TU.supercontig_111.6 | AT3G47600.1 | Myb domain protein 94 | 2.70301 | 15.5779 | K2 |
| evm.TU.supercontig_55.139 | AT4G12020.2 | Protein kinase family protein | 46.4331 | 224.131 | K2 |
| evm.TU.supercontig_53.101 | AT4G12020.2 | Protein kinase family protein | 14.3196 | 71.162 | K2 |
| evm.TU.supercontig_1195.3 | AT1G29860.1 | WRKY DNA-binding protein 71 | 0.903709 | 7.92522 | K2 |
| evm.TU.supercontig_31.71 | AT1G75250.1 | RAD-like 6 | 32.9336 | 156.964 | K2 |
| evm.TU.supercontig_1058.2 | AT5G04760.1 | Duplicated homeodomain-like superfamily protein | 106.098 | 495.931 | K2 |
| evm.TU.supercontig_62.11 | AT4G34410.1 | Redox responsive transcription factor 1 | 45.5797 | 227.128 | K2 |
| evm.TU.supercontig_224.11 | AT2G41690.1 | Heat shock transcription factor B3 | 13.4736 | 68.6939 | K2 |
| evm.TU.supercontig_21.142 | AT4G37260.1 | Myb domain protein 73 | 63.9986 | 312.307 | K2 |
| evm.TU.supercontig_1379.1 | AT3G01470.1 | Homeobox 1 | 1.98849 | 13.4868 | K2 |
| evm.TU.supercontig_864.1 | AT4G12020.3 | Protein kinase family protein | 86.1065 | 435.06 | K2 |
| evm.TU.supercontig_62.85 | AT5G49200.1 | WD-40 repeat family protein / zfwd4 protein (ZFWD4) | 7.49035 | 41.6703 | K2 |
| evm.TU.supercontig_11.111 | AT2G38250.1 | Homeodomain-like superfamily protein | 2.83535 | 18.2849 | K2 |
| evm.TU.supercontig_78.20 | AT4G12020.3 | Protein kinase family protein | 12.8638 | 67.9305 | K2 |
| evm.TU.contig_32256.1 | AT2G46400.1 | WRKY DNA-binding protein 46 | 100.229 | 501.127 | K2 |
| evm.TU.supercontig_2.219 | AT4G12020.2 | Protein kinase family protein | 38.8979 | 208.565 | K2 |
| evm.TU.contig_30912.1 | AT2G28710.1 | C2H2-type zinc finger family protein | 1.53354 | 12.3461 | K2 |
| evm.TU.supercontig_578.2 | AT4G12020.2 | Protein kinase family protein | 15.3704 | 137.273 | K3 |
| evm.TU.supercontig_55.154 | AT2G33710.1 | Integrase-type DNA-binding superfamily protein | 1.01697 | 15.8862 | K3 |
| evm.TU.supercontig_96.45 | AT2G28500.1 | LOB domain-containing protein 11 | 6.45897 | 62.6016 | K3 |
| evm.TU.supercontig_145.21 | AT2G47190.1 | Myb domain protein 2 | 41.5383 | 363.428 | K3 |
| evm.TU.supercontig_67.57 | AT3G12910.1 | NAC (No Apical Meristem) domain transcriptional regulator superfamily protein | 2.19042 | 28.8537 | K3 |
| evm.TU.supercontig_5.242 | AT2G38470.1 | WRKY DNA-binding protein 33 | 44.8722 | 421.063 | K3 |
| evm.TU.supercontig_101.16 | AT2G44840.1 | Ethylene-responsive element binding factor 13 | 12.759 | 131.366 | K3 |
| evm.TU.supercontig_50.56 | AT5G45710.1 | Winged-helix DNA-binding transcription factor family protein | 7.74852 | 96.4934 | K3 |
| evm.TU.supercontig_70.106 | AT5G14000.1 | NAC domain containing protein 84 | 46.7068 | 489.497 | K3 |
| evm.TU.contig_30789.1 | AT4G12020.2 | Protein kinase family protein | 1.99043 | 29.7961 | K3 |
| evm.TU.supercontig_65.53 | AT4G12020.3 | Protein kinase family protein | 6.51706 | 1.20911 | K4 |
| evm.TU.supercontig_74.79 | AT1G63100.1 | GRAS family transcription factor | 6.17451 | 1.06829 | K4 |
| evm.TU.supercontig_33.164 | AT3G56850.1 | ABA-responsive element binding protein 3 | 7.01196 | 1.31693 | K4 |
| evm.TU.supercontig_78.68 | AT4G32730.2 | Homeodomain-like protein | 8.44941 | 1.84908 | K4 |
| evm.TU.supercontig_29.116 | AT1G12260.1 | NAC 007 | 7.38483 | 1.56875 | K4 |
| evm.TU.supercontig_688.1 | AT4G12020.2 | Protein kinase family protein | 3.97716 | 0.838908 | K4 |
| evm.TU.supercontig_8.249 | AT3G49950.1 | GRAS family transcription factor | 6.26718 | 1.54032 | K4 |
| evm.TU.supercontig_106.83 | AT4G12020.2 | Protein kinase family protein | 2.99348 | 0.419109 | K4 |
| evm.TU.supercontig_10.192 | AT5G45260.1 | Disease resistance protein (TIR-NBS-LRR class) | 6.89299 | 1.63555 | K4 |
| evm.TU.supercontig_5.96 | AT4G12020.3 | Protein kinase family protein | 30.5912 | 7.07972 | K4 |
| evm.TU.supercontig_27.17 | AT1G28360.1 | ERF domain protein 12 | 26.6438 | 6.14588 | K4 |
| evm.TU.supercontig_2.281 | AT2G40620.1 | Basic-leucine zipper (bZIP) transcription factor family protein | 19.3864 | 4.3073 | K4 |
| evm.TU.supercontig_6.39 | AT3G49940.1 | LOB domain-containing protein 38 | 51.6767 | 11.6196 | K4 |
| evm.TU.supercontig_19.57 | AT4G12020.2 | Protein kinase family protein | 15.2849 | 2.91643 | K4 |
| evm.TU.supercontig_62.153 | AT4G39250.1 | RAD-like 1 | 811.05 | 200.301 | K4 |
| evm.TU.contig_34071.1 | AT5G62940.1 | Dof-type zinc finger DNA-binding family protein | 12.1363 | 2.27935 | K4 |
| evm.TU.supercontig_92.1 | AT4G12020.2 | Protein kinase family protein | 23.0878 | 4.49445 | K4 |
| evm.TU.supercontig_1729.1 | AT2G22840.1 | Growth-regulating factor 1 | 23.3387 | 4.5744 | K4 |
| evm.TU.supercontig_29.134 | AT2G29660.1 | Zinc finger (C2H2 type) family protein | 18.3347 | 3.17347 | K4 |
| evm.TU.supercontig_209.9 | AT3G58120.1 | Basic-leucine zipper (bZIP) transcription factor family protein | 15.1098 | 2.47565 | K4 |
| evm.TU.supercontig_80.88 | AT3G15270.1 | Squamosa promoter binding protein-like 5 | 29.3258 | 5.43142 | K4 |
| evm.TU.supercontig_19.100 | AT5G10570.1 | Basic helix-loop-helix (bHLH) DNA-binding superfamily protein | 3.68765 | 0 | K4 |
| evm.TU.supercontig_66.15 | AT2G02540.1 | Homeobox protein 21 | 8.77469 | 1.17127 | K4 |
| evm.TU.contig_32212.1 | AT4G12020.2 | Protein kinase family protein | 29.0695 | 5.60042 | K4 |
| evm.TU.supercontig_5.229 | AT4G12020.2 | Protein kinase family protein | 9.76126 | 0.813033 | K4 |
| evm.TU.supercontig_259.2 | AT5G25390.2 | Integrase-type DNA-binding superfamily protein | 15.8142 | 1.79703 | K4 |
| evm.TU.supercontig_1868.3 | AT4G12020.2 | Protein kinase family protein | 14.1291 | 1.28747 | K4 |
| evm.TU.supercontig_83.75 | AT1G03790.1 | Zinc finger C-x8-C-x5-C-x3-H type family protein | 32.0187 | 2.76801 | K4 |
| evm.TU.supercontig_14.16 | AT3G28857.1 | Basic helix-loop-helix (bHLH) DNA-binding family protein | 6.75247 | 0 | K4 |
| evm.TU.contig_24967.3 | AT4G12020.2 | Protein kinase family protein | 29.8976 | 1.57154 | K4 |

**Table S5.** The 59 differentially expressed genes encoding transporter proteins (TPs) between two cultivars.

| **Gene ID** | ***Arabidopsis*** | **Gene annotation** | **Expression level (FPKM)** | | **Cluster** |
| --- | --- | --- | --- | --- | --- |
|  |  | **TPs** | **SS-NP** | **SU-NP** |  |
| evm.TU.supercontig_3.5 | AT5G52860 | ABC-2 type transporter family protein | 45.0057 | 2.33714 | K1 |
| evm.TU.supercontig_161.4 | AT5G12380 | Annexin 8 | 5.04571 | 0.719332 | K2 |
| evm.TU.supercontig_575.2 | AT3G10600 | Cationic amino acid transporter 7 | 21.7472 | 5.20438 | K2 |
| evm.TU.supercontig_37.14 | AT5G09220 | Amino acid permease 2 | 4.1724 | 0.395619 | K2 |
| evm.TU.supercontig_37.163 | AT2G21050 | Like AUXIN RESISTANT 2 | 21.1376 | 4.7921 | K2 |
| evm.TU.supercontig_14.259 | AT3G30390 | Transmembrane amino acid transporter family protein | 11.5874 | 2.32298 | K2 |
| evm.TU.supercontig_190.36 | AT4G01470 | Tonoplast intrinsic protein 1;3 | 139.781 | 22.6553 | K2 |
| evm.TU.supercontig_336.2 | AT5G60020 | Laccase 17 | 9.69436 | 1.4099 | K2 |
| evm.TU.supercontig_163.26 | AT2G37460 | Nodulin MtN21 /EamA-like transporter family protein | 33.939 | 6.9996 | K2 |
| evm.TU.supercontig_26.308 | AT5G03260 | Laccase 11 | 22.893 | 3.84154 | K2 |
| evm.TU.supercontig_109.27 | AT4G17340 | Tonoplast intrinsic protein 2;2 | 694.378 | 139.609 | K2 |
| evm.TU.supercontig_103.53 | AT5G21105 | Plant L-ascorbate oxidase | 15.2722 | 2.12329 | K2 |
| evm.TU.supercontig_69.87 | AT1G12940 | Nitrate transporter2.5 | 2.67323 | 66.8621 | K3 |
| evm.TU.supercontig_307.1 | AT3G12750 | Zinc transporter 1 precursor | 3.58291 | 62.3305 | K3 |
| evm.TU.supercontig_163.8 | AT2G38100 | Proton-dependent oligopeptide transport (POT) family protein | 26.399 | 356.769 | K3 |
| evm.TU.supercontig_6.348 | AT2G17500 | Auxin efflux carrier family protein | 18.3344 | 199.704 | K3 |
| evm.TU.contig_36671.1 | AT5G12380 | Annexin 8 | 7.06387 | 90.9406 | K3 |
| evm.TU.supercontig_97.97 | AT3G13080 | Multidrug resistance-associated protein 3 | 2.18211 | 23.0908 | K3 |
| evm.TU.supercontig_5.274 | AT1G69870 | Nitrate transporter 1.7 | 25.9909 | 203.961 | K3 |
| evm.TU.supercontig_96.2 | AT5G53130 | Cyclic nucleotide gated channel 1 | 11.6095 | 93.1071 | K3 |
| evm.TU.supercontig_10.73 | AT1G15960 | NRAMP metal ion transporter 6 | 1.76615 | 19.476 | K3 |
| evm.TU.supercontig_1109.2 | AT1G12600 | UDP-N-acetylglucosamine (UAA) transporter family | 2.04974 | 24.1666 | K3 |
| evm.TU.contig_35659.1 | AT4G01010 | Cyclic nucleotide-gated channel 13 | 5.86577 | 60.6668 | K3 |
| evm.TU.supercontig_8.267 | AT1G05300 | Zinc transporter 5 precursor | 0 | 2.03021 | K4 |
| evm.TU.supercontig_43.2 | AT1G04120 | Multidrug resistance-associated protein 5 | 1.7926 | 7.33044 | K4 |
| evm.TU.supercontig_50.169 | AT5G49130 | MATE efflux family protein | 0.425286 | 2.67985 | K4 |
| evm.TU.supercontig_23.40 | AT2G29120 | Glutamate receptor 2.7 | 0.320559 | 2.17406 | K4 |
| evm.TU.supercontig_2.38 | AT1G25270 | Nodulin MtN21 /EamA-like transporter family protein | 0.202419 | 1.85923 | K4 |
| evm.TU.supercontig_6.339 | AT1G76490 | Hydroxy methylglutaryl CoA reductase 1 | 0.373307 | 2.02832 | K4 |
| evm.TU.supercontig_1.293 | AT3G55090 | ABC-2 type transporter family protein | 0.468007 | 2.19403 | K4 |
| evm.TU.supercontig_28.77 | AT5G12080 | Mechanosensitive channel of small conductance-like 10 | 9.12271 | 61.9686 | K4 |
| evm.TU.supercontig_30.98 | AT3G12750 | Zinc transporter 1 precursor | 3.50902 | 25.8696 | K4 |
| evm.TU.supercontig_19.10 | AT1G66950 | Pleiotropic drug resistance 11 | 7.24251 | 46.3446 | K4 |
| evm.TU.supercontig_10.181 | AT1G15460 | HCO3- transporter family | 2.88164 | 21.3624 | K4 |
| evm.TU.supercontig_33.12 | AT1G23300 | MATE efflux family protein | 21.1118 | 122.4 | K4 |
| evm.TU.supercontig_19.9 | AT2G36380 | Pleiotropic drug resistance 6 | 4.99586 | 32.2541 | K4 |
| evm.TU.supercontig_7.165 | AT1G60960 | Iron regulated transporter 3 | 18.413 | 104.046 | K4 |
| evm.TU.supercontig_120.27 | AT2G38290 | Ammonium transporter 2 | 20.9747 | 117.723 | K4 |
| evm.TU.supercontig_36.190 | AT1G28220 | Purine permease 3 | 11.1686 | 62.102 | K4 |
| evm.TU.contig_31100.1 | AT1G08920 | ERD (early response to dehydration) six-like 1 | 2.54556 | 17.2646 | K4 |
| evm.TU.supercontig_9.188 | AT4G13420 | High affinity K+ transporter 5 | 1.85622 | 9.01481 | K4 |
| evm.TU.supercontig_46.12 | AT1G02630 | Nucleoside transporter family protein | 2.30106 | 10.125 | K4 |
| evm.TU.supercontig_447.2 | AT5G18840 | Major facilitator superfamily protein | 2.44747 | 12.4725 | K4 |
| evm.TU.supercontig_43.99 | AT5G16740 | Transmembrane amino acid transporter family protein | 1.30498 | 8.02731 | K4 |
| evm.TU.supercontig_112.55 | AT5G26250 | Major facilitator superfamily protein | 2.09299 | 10.296 | K4 |
| evm.TU.supercontig_14.218 | AT1G28220 | Purine permease 3 | 4.88557 | 20.8965 | K4 |
| evm.TU.contig_43318.1 | AT3G02690 | Nodulin MtN21 /EamA-like transporter family protein | 0 | 2.72852 | K4 |
| evm.TU.supercontig_34.100 | AT4G03320 | Translocon at the inner envelope membrane of chloroplasts 20-IV | 0.611152 | 6.4647 | K4 |
| evm.TU.supercontig_603.3 | AT1G15520 | Pleiotropic drug resistance 12 | 1.01202 | 8.00757 | K4 |
| evm.TU.supercontig_5.164 | AT2G41190 | Transmembrane amino acid transporter family protein | 1.96954 | 12.1656 | K4 |
| evm.TU.supercontig_235.1 | AT5G19640 | Major facilitator superfamily protein | 1.9905 | 12.0939 | K4 |
| evm.TU.supercontig_135.39 | AT1G31260 | Zinc transporter 10 precursor | 17.8701 | 80.926 | K4 |
| evm.TU.supercontig_1046.1 | AT5G07050 | Nodulin MtN21 /EamA-like transporter family protein | 8.58251 | 40.2199 | K4 |
| evm.TU.supercontig_52.122 | AT3G63380 | ATPase E1-E2 type family protein / haloacid dehalogenase-like hydrolase family protein | 10.7931 | 47.161 | K4 |
| evm.TU.supercontig_36.45 | AT4G08300 | Nodulin MtN21 /EamA-like transporter family protein | 23.3313 | 98.6003 | K4 |
| evm.TU.supercontig_321.1 | AT3G18440 | Aluminum-activated malate transporter 9 | 5.75214 | 26.8163 | K4 |
| evm.TU.supercontig_36.44 | AT4G08290 | Nodulin MtN21 /EamA-like transporter family protein | 2.1597 | 12.1444 | K4 |
| evm.TU.supercontig_73.21 | AT1G33110 | MATE efflux family protein | 25.2241 | 109.06 | K4 |
| evm.TU.contig_29648.1 | AT5G40780 | Lysine histidine transporter 1 | 3.34834 | 17.3872 | K4 |

**Table S6.** The 66 differentially expressed hormone-related genes between two cultivars.

| **Gene ID** | ***Arabidopsis*** | **Gene annotation** | **Related Hormone** | **Expression level (FPKM)** | | **Cluster** |
| --- | --- | --- | --- | --- | --- | --- |
|  |  |  |  | **SS-NP** | **SU-NP** |  |
| evm.TU.supercontig_12.204 | AT5G55540 | Tornado 1 | auxin | 4.39367 | 0.661513 | K1 |
| evm.TU.supercontig_390.6 | AT1G80080 | Leucine-rich repeat (LRR) family protein | abscisic acid | 3.4106 | 0.471965 | K1 |
| evm.TU.supercontig_9.199 | AT2G47750 | Putative indole-3-acetic acid-amido synthetase GH3.9 | auxin | 4.542 | 0.953457 | K1 |
| evm.TU.supercontig_12.105 | AT3G44730 | Kinesin-like protein 1 | salicylic acid | 4.65776 | 0.973752 | K1 |
| evm.TU.supercontig_21.121 | AT2G39540 | Gibberellin-regulated family protein | gibberellin | 153.908 | 34.6792 | K1 |
| evm.TU.supercontig_7.98 | AT4G18780 | Cellulose synthase family protein | abscisic acid | 5.82756 | 0.818032 | K1 |
| evm.TU.supercontig_2179.1 | AT1G02205 | Fatty acid hydroxylase superfamily | abscisic acid | 6.82184 | 1.10752 | K1 |
| evm.TU.supercontig_37.203 | AT4G34800 | SAUR-like auxin-responsive protein family | auxin | 2.66234 | 0 | K1 |
| evm.TU.supercontig_12.205 | AT5G55540 | Tornado 1 | auxin | 6.52791 | 1.09295 | K1 |
| evm.TU.supercontig_46.172 | AT4G30610 | Alpha/beta-Hydrolases superfamily protein | brassinosteroid | 9.76337 | 1.77575 | K1 |
| evm.TU.supercontig_27.17 | AT1G28360 | ERF domain protein 12 | ethylene | 26.6438 | 6.14588 | K1 |
| evm.TU.supercontig_36.151 | AT4G08950 | Phosphate-responsive 1 family protein | brassinosteroid | 11.8528 | 2.35711 | K1 |
| evm.TU.supercontig_109.12 | AT2G20000 | CDC27 family protein | auxin | 7.33951 | 1.17736 | K1 |
| evm.TU.supercontig_27.36 | AT2G33790 | Arabinogalactan protein 30 | abscisic acid | 126.905 | 10.0667 | K2 |
| evm.TU.supercontig_200.13 | AT4G34160 | CYCLIN D3;1 | brassinosteroid cytokinin | 27.0426 | 1.68664 | K2 |
| evm.TU.supercontig_14.88 | AT5G14920 | Gibberellin-regulated family protein | gibberellin | 33.3247 | 2.15195 | K2 |
| evm.TU.supercontig_37.215 | AT5G18060 | SAUR-like auxin-responsive protein family | auxin | 4.97534 | 0 | K2 |
| evm.TU.supercontig_41.42 | AT1G02205 | Fatty acid hydroxylase superfamily | abscisic acid | 184.548 | 24.8403 | K2 |
| evm.TU.contig_37833.1 | AT5G20820 | SAUR-like auxin-responsive protein family | auxin | 24.7374 | 2.89335 | K2 |
| evm.TU.supercontig_157.25 | AT5G59310 | Lipid transfer protein 4 | abscisic acid | 83.6708 | 1225.39 | K3 |
| evm.TU.supercontig_50.27 | AT3G23230 | Integrase-type DNA-binding superfamily protein | ethylene | 3.81205 | 79.7241 | K3 |
| evm.TU.supercontig_157.27 | AT5G59310 | Lipid transfer protein 4 | abscisic acid | 23.3339 | 433.616 | K3 |
| evm.TU.supercontig_34.60 | AT1G34670 | Myb domain protein 93 | abscisic acid auxin salicylic acid | 26.4289 | 555.113 | K3 |
| evm.TU.supercontig_104.69 | AT1G52340 | NAD(P)-binding Rossmann-fold superfamily protein | abscisic acid | 0.561051 | 28.8165 | K3 |
| evm.TU.contig_47211.1 | AT5G24860 | Flowering promoting factor 1 | gibberellin | 0 | 30.678 | K3 |
| evm.TU.supercontig_6.348 | AT2G17500 | Auxin efflux carrier family protein | auxin | 18.3344 | 199.704 | K4 |
| evm.TU.supercontig_50.56 | AT5G45710 | Winged-helix DNA-binding transcription factor family protein | auxin ethylene | 7.74852 | 96.4934 | K4 |
| evm.TU.supercontig_5.242 | AT2G38470 | WRKY DNA-binding protein 33 | abscisic acid | 44.8722 | 421.063 | K4 |
| evm.TU.supercontig_101.16 | AT2G44840 | Ethylene-responsive element binding factor 13 | ethylene | 12.759 | 131.366 | K4 |
| evm.TU.supercontig_145.21 | AT2G47190 | Myb domain protein 2 | abscisic acid ethylene salicylic acid | 41.5383 | 363.428 | K4 |
| evm.TU.supercontig_23.98 | AT3G24500 | Multiprotein bridging factor 1C | ethylene abscisic acid | 21.5776 | 176.415 | K4 |
| evm.TU.contig_34243.1 | AT2G29420 | Glutathione S-transferase tau 7 | salicylic acid | 29.2826 | 234.884 | K4 |
| evm.TU.supercontig_62.9 | AT3G19270 | Cytochrome P450, family 707, subfamily A, polypeptide 4 | abscisic acid | 0.659086 | 2.71614 | K4 |
| evm.TU.supercontig_544.1 | AT2G18470 | Roline-rich extensin-like receptor kinase 4 | abscisic acid | 0.454824 | 2.46352 | K4 |
| evm.TU.supercontig_49.19 | AT3G19270 | Cytochrome P450, family 707, subfamily A, polypeptide 4 | abscisic acid | 1.92756 | 9.48698 | K4 |
| evm.TU.supercontig_46.12 | AT1G02630 | Nucleoside transporter family protein | cytokinin | 2.30106 | 10.125 | K4 |
| evm.TU.supercontig_5.28 | AT2G36270 | Basic-leucine zipper (bZIP) transcription factor family protein | abscisic acid | 0.696095 | 3.65288 | K4 |
| evm.TU.supercontig_43.2 | AT1G04120 | Multidrug resistance-associated protein 5 | abscisic acid | 1.7926 | 7.33044 | K4 |
| evm.TU.supercontig_3.494 | AT1G68320 | Myb domain protein 62 | salicylic acid | 23.1542 | 170.373 | K4 |
| evm.TU.supercontig_48.133 | AT5G26920 | Cam-binding protein 60-like G | salicylic acid | 49.9857 | 327.068 | K4 |
| evm.TU.supercontig_3.54 | AT4G23810 | WRKY family transcription factor | salicylic acid | 67.3238 | 442.734 | K4 |
| evm.TU.supercontig_59.60 | AT2G29420 | Glutathione S-transferase tau 7 | salicylic acid | 29.6253 | 202.733 | K4 |
| evm.TU.supercontig_2.408 | AT2G30020 | Protein phosphatase 2C family protein | abscisic acid | 59.7727 | 403.526 | K4 |
| evm.TU.supercontig_74.83 | AT4G25480 | Dehydration response element B1A | salicylic acid | 11.6146 | 75.5762 | K4 |
| evm.TU.supercontig_3.487 | AT4G21410 | Cysteine-rich RLK (RECEPTOR-like protein kinase) 29 | abscisic acid | 2.49472 | 19.0917 | K4 |
| evm.TU.supercontig_129.23 | AT5G65670 | Indole-3-acetic acid inducible 9 | auxin | 49.3207 | 288.859 | K4 |
| evm.TU.supercontig_14.99 | AT5G61430 | NAC domain containing protein 100 | brassinosteroid | 6.85613 | 43.7501 | K4 |
| evm.TU.contig_32826.1 | AT5G54510 | Auxin-responsive GH3 family protein | auxin | 0.416734 | 7.07705 | K4 |
| evm.TU.supercontig_19.158 | AT2G30020 | Protein phosphatase 2C family protein | abscisic acid | 10.2035 | 46.3972 | K4 |
| evm.TU.supercontig_14.83 | AT3G28210 | Zinc finger (AN1-like) family protein | abscisic acid | 26.1943 | 115.174 | K4 |
| evm.TU.supercontig_50.20 | AT2G31180 | Myb domain protein 14 | auxin ethylene jasmonic acid salicylic acid | 109.381 | 445.607 | K4 |
| evm.TU.supercontig_62.84 | AT2G23460 | Extra-large G-protein 1 | abscisic acid | 39.8324 | 161.894 | K4 |
| evm.TU.supercontig_133.24 | AT2G32440 | Ent-kaurenoic acid hydroxylase 2 | gibberellin | 0 | 3.01436 | K4 |
| evm.TU.supercontig_3.146 | AT2G46690 | SAUR-like auxin-responsive protein family | auxin | 25.0846 | 106.423 | K4 |
| evm.TU.supercontig_111.23 | AT5G61430 | NAC domain containing protein 100 | brassinosteroid | 8.31575 | 37.3013 | K4 |
| evm.TU.supercontig_83.80 | AT5G44210 | Erf domain protein 9 | ethylene | 70.8987 | 323.625 | K4 |
| evm.TU.supercontig_36.152 | AT4G08950 | Phosphate-responsive 1 family protein | brassinosteroid | 238.645 | 1084.21 | K4 |
| evm.TU.supercontig_190.34 | AT3G46130 | Myb domain protein 48 | abscisic acid ethylene gibberellin jasmonic acid salicylic acid | 1.53937 | 10.5484 | K4 |
| evm.TU.supercontig_603.3 | AT1G15520 | Pleiotropic drug resistance 12 | ethylene jasmonic acid salicylic acid | 1.01202 | 8.00757 | K4 |
| evm.TU.supercontig_111.6 | AT3G47600 | Myb domain protein 94 | abscisic acid auxin ethylene jasmonic acid salicylic acid | 2.70301 | 15.5779 | K4 |
| evm.TU.supercontig_87.67 | AT2G29420 | Glutathione S-transferase tau 7 | salicylic acid | 7.722 | 42.059 | K4 |
| evm.TU.supercontig_28.53 | AT2G06050 | Oxophytodienoate-reductase 3 | gibberellin jasmonic acid | 42.0522 | 206.467 | K4 |
| evm.TU.supercontig_21.142 | AT4G37260 | Myb domain protein 73 | abscisic acid auxin ethylene gibberellin jasmonic acid salicylic acid | 63.9986 | 312.307 | K4 |
| evm.TU.supercontig_59.106 | AT5G13930 | Chalcone and stilbene synthase family protein | auxin jasmonic acid | 0.679867 | 7.5339 | K4 |
| evm.TU.supercontig_36.153 | AT4G08950 | Phosphate-responsive 1 family protein | brassinosteroid | 129.146 | 664.857 | K4 |
| evm.TU.contig_31100.1 | AT1G08920 | ERD (early response to dehydration) six-like 1 | abscisic acid | 2.54556 | 17.2646 | K4 |

**Table S7.** A total of 21 candidate DEGs (in red) were selected for qRT-PCR analysis.

| **Gene ID** | **Arabidopsis** | **Gene annotation** | **FPKM** | | **log_2_(SU-NP/SS-NP)** | ***p*-value** | **FDR (ajusted *p*-value)** |
| --- | --- | --- | --- | --- | --- | --- | --- |
|  |  |  | **SS-NP** | **SU-NP** |  |  |  |
| GO-enrichment No.1:microtubule-based movement (1/13) | | |  |  |  |  |  |
| evm.TU.supercontig_81.9 | AT4G21270.1 | Kinesin 1 | 7.91875 | 0.546433 | -3.85716 | 5.00E-05 | 0.000642247 |
| KEGG-enrichment No.1:Phenylpropanoid biosynthesis (4/11) | | |  |  |  |  |  |
| evm.TU.supercontig_8.226 | AT1G05260.1 | Peroxidase superfamily protein | 22.8076 | 4.47048 | -2.35101 | 5.00E-05 | 0.000642247 |
| evm.TU.supercontig_2.159 | AT1G67980.1 | Caffeoyl-CoA 3-O-methyltransferase | 2.79823 | 14.7322 | 2.39639 | 5.00E-05 | 0.000642247 |
| evm.TU.supercontig_152.14 | AT4G21960.1 | Peroxidase superfamily protein | 813.054 | 58.6159 | -3.79399 | 5.00E-05 | 0.000642247 |
| evm.TU.supercontig_1127.3 | AT5G06730.1 | Peroxidase superfamily protein | 2.15102 | 16.0108 | 2.89595 | 5.00E-05 | 0.000642247 |
| GO terms which only contain up-regulated genes (5/16) | | |  |  |  |  |  |
| evm.TU.supercontig_112.47 | AT5G58860.1 | Cytochrome P450, family 86, subfamily A, polypeptide 1 | 0 | 3.54267 | inf | 5.00E-05 | 0.000642247 |
| evm.TU.supercontig_7.185 | AT4G33040.1 | Thioredoxin superfamily protein | 43.2834 | 410.656 | 3.24605 | 5.00E-05 | 0.000642247 |
| evm.TU.supercontig_55.91 | AT1G10630.1 | ADP-ribosylation factor A1F | 39.9813 | 202.785 | 2.34255 | 5.00E-05 | 0.000642247 |
| evm.TU.contig_cp_cp |  |  | 0 | 23.1326 | inf | 5.00E-05 | 0.000642247 |
| evm.TU.contig_47264.1 |  |  | 0 | 115.232 | inf | 5.00E-05 | 0.000642247 |
| GO terms which only contain down-regulated genes (3/4) | | |  |  |  |  |  |
| evm.TU.supercontig_19.250 | AT5G05940.1 | ROP guanine nucleotide exchange factor 5 | 7.62501 | 1.75033 | -2.12311 | 5.00E-05 | 0.000642247 |
| evm.TU.supercontig_27.36 | AT2G33790.1 | Arabinogalactan protein 30 | 126.905 | 10.0667 | -3.65609 | 5.00E-05 | 0.000642247 |
| evm.TU.supercontig_8.254 | AT3G22142.1 | Bifunctional inhibitor/lipid-transfer protein/seed storage 2S albumin superfamily protein | 616.123 | 79.416 | -2.95572 | 5.00E-05 | 0.000642247 |
| Candidate DEGs annotated to transcription factors (TFs) (8/118) | | |  |  |  |  |  |
| evm.TU.supercontig_152.35 | AT1G62300.1 | WRKY family transcription factor | 22.0205 | 136.961 | 2.63685 | 5.00E-05 | 0.000642247 |
| evm.TU.supercontig_9.35 | AT5G49520.1 | WRKY DNA-binding protein 48 | 17.2988 | 106.025 | 2.61567 | 5.00E-05 | 0.000642247 |
| evm.TU.supercontig_126.44 | AT5G26170.1 | WRKY DNA-binding protein 50 | 3.85459 | 99.2079 | 4.6858 | 5.00E-05 | 0.000642247 |
| evm.TU.supercontig_190.34 | AT3G46130.1 | Myb domain protein 48 | 1.53937 | 10.5484 | 2.77661 | 0.00065 | 0.00556837 |
| evm.TU.supercontig_111.6 | AT3G47600.1 | Myb domain protein 94 | 2.70301 | 15.5779 | 2.52687 | 5.00E-05 | 0.000642247 |
| evm.TU.supercontig_3.494 | AT1G68320.1 | Myb domain protein 62 | 23.1542 | 170.373 | 2.87935 | 5.00E-05 | 0.000642247 |
| evm.TU.supercontig_12.9 | AT4G27300.1 | S-locus lectin protein kinase family protein | 3.27164 | 26.05 | 2.9932 | 5.00E-05 | 0.000642247 |
| evm.TU.supercontig_10.192 | AT3G14470.1 | NB-ARC domain-containing disease resistance protein | 6.89299 | 1.63555 | -2.07535 | 5.00E-05 | 0.000642247 |

**Table S8.** Details of oligonucleotide primers used for qRT-PCR.

| Gene Name | Forward Name | Forward | Start | End | Length | Reverse Name | Reverse | Start | End | Length | Amplicon Length |
| --- | --- | --- | --- | --- | --- | --- | --- | --- | --- | --- | --- |
| evm.TU.supercontig_10.192 | S10.192-F | GCAGGAAGACGAGATACAAGTC | 1556 | 1578 | 22 | S10.192-R | AGAAGGAGGGTGCGTAAGTA | 1642 | 1662 | 20 | 106 |
| evm.TU.supercontig_111.6 | S111.6-F | ACGCATCCAGCACAGAAA | 569 | 587 | 18 | S111.6-R | GCCATCCTGGTGTGAAGATTA | 651 | 672 | 21 | 103 |
| evm.TU.supercontig_112.47 | S112.47-F | CGAACCTCCGAAAGATGGATAC | 1311 | 1333 | 22 | S112.47-R | CAGAGGCCACAGACTTCATTT | 1394 | 1415 | 21 | 104 |
| evm.TU.supercontig_1127.3 | S1127.3-F | GTTGCCCTAATGTGACGAGTAT | 104 | 126 | 22 | S1127.3-R | CGAAGCAATCGTGAAAGTGAAG | 190 | 212 | 22 | 108 |
| evm.TU.supercontig_12.9 | S12.9-F | GCTGAGCCAAACAGACAGA | 16 | 35 | 19 | S12.9-R | TGCAAGATCCATCCGAATACC | 96 | 117 | 21 | 101 |
| evm.TU.supercontig_126.44 | S126.44-F | GAAGATGAATGGGTTAGGGTAGAA | 76 | 100 | 24 | S126.44-R | GGTTGAGTTGGGATGTGAAGA | 147 | 168 | 21 | 92 |
| evm.TU.supercontig_152.14 | S152.14-F | CTGCCAAAGCTCTCTTCTTCT | 5 | 26 | 21 | S152.14-R | GGTTTCCTCCTTCCTCATTCTC | 64 | 86 | 22 | 81 |
| evm.TU.supercontig_152.35 | S152.35-F | GATCGGAAGCTCCCATGATTAC | 1061 | 1083 | 22 | S152.35-R | AGCACGAGGACAAGGATTTC | 1127 | 1147 | 20 | 86 |
| evm.TU.supercontig_19.250 | S19.25-F | CCAGCTCTGCGTAAACTAGAC | 613 | 634 | 21 | S19.25-R | CTGGTGCTACAATCCCTTGAT | 689 | 710 | 21 | 97 |
| evm.TU.supercontig_190.34 | S190.34-F | GGTGCAAGAGGAAACCAGAA | 3 | 23 | 20 | S190.34-R | CCCATCGTCGATCTCCAAATAA | 73 | 95 | 22 | 92 |
| evm.TU.supercontig_2.159 | S2.159-F | GGGTGTACCAGTTGATGAAGG | 147 | 168 | 21 | S2.159-R | GCAGTGGCCAAGAGAGAATAG | 237 | 258 | 21 | 111 |
| evm.TU.supercontig_27.36 | S27.36-F | ATACCCTCTTGGGAGCTTCA | 410 | 430 | 20 | S27.36-R | GTAGCCGTTCTTGTCTGTCTTC | 495 | 517 | 22 | 107 |
| evm.TU.supercontig_3.494 | S3.494-F | GTAAGCGGTAGTTCCAGTGAAG | 22 | 44 | 22 | S3.494-R | CACCGTGGCAGGAGATATAATG | 106 | 128 | 22 | 106 |
| evm.TU.supercontig_55.91 | S55.91-F | GGATGTAGGAGGCCAAGATAAG | 147 | 169 | 22 | S55.91-R | TTGCTGTCCACCACAAAGA | 215 | 234 | 19 | 87 |
| evm.TU.supercontig_7.185 | S7.185-F | CGGAGCATCCTGTAATCATCTT | 125 | 147 | 22 | S7.185-R | CGATCTCGTCCTCGTCTAACT | 224 | 245 | 21 | 120 |
| evm.TU.supercontig_8.226 | S8.226-F | ATCCTCCACAACCCTCTCTTA | 822 | 843 | 21 | S8.226-R | TTGACATTGACTCCTCCCATTT | 908 | 930 | 22 | 108 |
| evm.TU.supercontig_8.254 | S8.254-F | CCTTTGTCTCTGCACCACTATTA | 657 | 680 | 23 | S8.254-R | CTTCCCGCAGTCAATGAGAA | 728 | 748 | 20 | 91 |
| evm.TU.supercontig_81.9 | S81.9-F | GTGTCAACACCATTCCCTTTG | 56 | 77 | 21 | S81.9-R | GGACGAACCATCTTGCTTATTG | 138 | 160 | 22 | 104 |
| evm.TU.supercontig_9.35 | S9.35-F | TTTCCGACGAGGCTACTTTG | 68 | 88 | 20 | S9.35-R | CCCGAACCAGAACCAGAATAA | 141 | 162 | 21 | 94 |
| evm.TU.contig_47264.1 | C47264.1-F | GCCCAGCTATCTGTCACTTTA | 233 | 254 | 21 | C47264.1-R | CAACGATGGCCTTTCCTTTATC | 304 | 326 | 22 | 93 |
| evm.TU.contig_cp_cp | cp-F | TTACTAACACTGCCGTCCATAC | 80 | 102 | 22 | cp-R | GCTAGATACGCTTTCGACTTCT | 195 | 217 | 22 | 137 |

**Table S9.** The normalized expression level (FPKM) of RNA-seq and the relative qRT-PCR expression level for the 21 selected DEGs.

| Gene ID | Expression Level (RNA-seq) | | | Expression Level (qRT-PCR) | | |
| --- | --- | --- | --- | --- | --- | --- |
|  | FPKM | | log_2_(SU-NP/SS-NP) | Relative Expression | | log_2_(SU-NP/SS-NP) |
|  | SS-NP | SU-NP |  | SS-NP | SU-NP |  |
| evm.TU.supercontig_10.192 | 6.8930 | 1.6356 | -2.0754 | 1.0000 | 0.0271 | -5.2063 |
| evm.TU.supercontig_111.6 | 2.7030 | 15.5779 | 2.5269 | 0.2633 | 1.0000 | 1.9254 |
| evm.TU.supercontig_112.47 | 0.0000 | 3.5427 | inf | 0.3779 | 1.0000 | 1.4038 |
| evm.TU.supercontig_1127.3 | 2.1510 | 16.0108 | 2.8960 | 0.6796 | 1.0000 | 0.5572 |
| evm.TU.supercontig_12.9 | 3.2716 | 26.0500 | 2.9932 | 0.2087 | 1.0000 | 2.2603 |
| evm.TU.supercontig_126.44 | 3.8546 | 99.2079 | 4.6858 | 0.3237 | 1.0000 | 1.6274 |
| evm.TU.supercontig_152.14 | 813.0540 | 58.6159 | -3.7940 | 1.0000 | 0.0050 | -7.6344 |
| evm.TU.supercontig_152.35 | 22.0205 | 136.9610 | 2.6369 | 0.4877 | 1.0000 | 1.0360 |
| evm.TU.supercontig_19.250 | 7.6250 | 1.7503 | -2.1231 | 1.0000 | 0.1824 | -2.4545 |
| evm.TU.supercontig_190.34 | 1.5394 | 10.5484 | 2.7766 | 0.5323 | 1.0000 | 0.9098 |
| evm.TU.supercontig_2.159 | 2.7982 | 14.7322 | 2.3964 | 0.5915 | 1.0000 | 0.7575 |
| evm.TU.supercontig_27.36 | 126.9050 | 10.0667 | -3.6561 | 1.0000 | 0.0050 | -7.6412 |
| evm.TU.supercontig_3.494 | 23.1542 | 170.3730 | 2.8794 | 0.1901 | 1.0000 | 2.3953 |
| evm.TU.supercontig_55.91 | 39.9813 | 202.7850 | 2.3426 | 0.1686 | 1.0000 | 2.5682 |
| evm.TU.supercontig_7.185 | 43.2834 | 410.6560 | 3.2461 | 0.0995 | 1.0000 | 3.3294 |
| evm.TU.supercontig_8.226 | 22.8076 | 4.4705 | -2.3510 | 1.0000 | 0.0574 | -4.1238 |
| evm.TU.supercontig_8.254 | 616.1230 | 79.4160 | -2.9557 | 1.0000 | 0.0083 | -6.9093 |
| evm.TU.supercontig_81.9 | 7.9188 | 0.5464 | -3.8572 | 1.0000 | 0.0042 | -7.8880 |
| evm.TU.supercontig_9.35 | 17.2988 | 106.0250 | 2.6157 | 0.1681 | 1.0000 | 2.5728 |
| evm.TU.contig_47264.1 | 0.0000 | 115.2320 | inf | 0.0123 | 1.0000 | 6.3475 |
| evm.TU.contig_cp_cp | 0.0000 | 23.1326 | inf | 0.0000 | 1.0000 | inf |

**Table S10a.** Level 2 Gene Ontology (GO) annotation of 610 genes showing Sunset AS exclusive events in three main ontologies: **a** Molecular function. **b** Biological process and **c** Cellular component.

Total gene number with GO ID: 261

| **Term type** | **Function description** | **No.** | **Percentage** | **GO** |
| --- | --- | --- | --- | --- |
| **Molecular function** | electron carrier activity | 3 | 1.15% | GO:0009055 |
|  | binding | 111 | 42.53% | GO:0005488 |
|  | transporter activity | 10 | 3.83% | GO:0005215 |
|  | catalytic activity | 131 | 50.19% | GO:0003824 |
|  | nutrient reservoir activity | 2 | 0.77% | GO:0045735 |
|  | enzyme regulator activity | 2 | 0.77% | GO:0030234 |
|  | structural molecule activity | 6 | 2.30% | GO:0005198 |
|  | nucleic acid binding transcription factor activity | 5 | 1.92% | GO:0001071 |
|  | antioxidant activity | 3 | 1.15% | GO:0016209 |
| **Cellular component** | virion part | 1 | 0.38% | GO:0044423 |
|  | symplast | 1 | 0.38% | GO:0055044 |
|  | extracellular matrix | 1 | 0.38% | GO:0031012 |
|  | organelle | 68 | 26.05% | GO:0043226 |
|  | cell part | 96 | 36.78% | GO:0044464 |
|  | membrane-enclosed lumen | 2 | 0.77% | GO:0031974 |
|  | virion | 1 | 0.38% | GO:0019012 |
|  | membrane part | 36 | 13.79% | GO:0044425 |
|  | extracellular region | 10 | 3.83% | GO:0005576 |
|  | cell junction | 1 | 0.38% | GO:0030054 |
|  | membrane | 53 | 20.31% | GO:0016020 |
|  | macromolecular complex | 29 | 11.11% | GO:0032991 |
|  | cell | 96 | 36.78% | GO:0005623 |
|  | organelle part | 25 | 9.58% | GO:0044422 |
| **Bological process** | reproduction | 1 | 0.38% | GO:0000003 |
|  | multi-organism process | 5 | 1.92% | GO:0051704 |
|  | developmental process | 19 | 7.28% | GO:0032502 |
|  | positive regulation of biological process | 3 | 1.15% | GO:0048518 |
|  | multicellular organismal process | 19 | 7.28% | GO:0032501 |
|  | cellular process | 134 | 51.34% | GO:0009987 |
|  | single-organism process | 116 | 44.44% | GO:0044699 |
|  | growth | 6 | 2.30% | GO:0040007 |
|  | signaling | 10 | 3.83% | GO:0023052 |
|  | negative regulation of biological process | 8 | 3.07% | GO:0048519 |
|  | metabolic process | 168 | 64.37% | GO:0008152 |
|  | biological regulation | 41 | 15.71% | GO:0065007 |
|  | reproductive process | 8 | 3.07% | GO:0022414 |
|  | immune system process | 3 | 1.15% | GO:0002376 |
|  | regulation of biological process | 37 | 14.18% | GO:0050789 |
|  | cellular component organization or biogenesis | 30 | 11.49% | GO:0071840 |
|  | response to stimulus | 30 | 11.49% | GO:0050896 |
|  | localization | 33 | 12.64% | GO:0051179 |

**Table S10b.** Level 2 Gene Ontology (GO) annotation of 611 genes showing SunUp AS exclusive events in three main ontologies: **a** Molecular function. **b** Biological process and **c** Cellular component.

Total gene number with GO ID: 280

| **Term type** | **Function description** | **No.** | **Percentage** | **GO** |
| --- | --- | --- | --- | --- |
| **Molecular function** | electron carrier activity | 2 | 0.71% | GO:0009055 |
|  | protein binding transcription factor activity | 1 | 0.36% | GO:0000988 |
|  | binding | 134 | 47.86% | GO:0005488 |
|  | transporter activity | 20 | 7.14% | GO:0005215 |
|  | catalytic activity | 150 | 53.57% | GO:0003824 |
|  | nutrient reservoir activity | 2 | 0.71% | GO:0045735 |
|  | enzyme regulator activity | 3 | 1.07% | GO:0030234 |
|  | molecular transducer activity | 2 | 0.71% | GO:0060089 |
|  | structural molecule activity | 3 | 1.07% | GO:0005198 |
|  | receptor activity | 1 | 0.36% | GO:0004872 |
|  | nucleic acid binding transcription factor activity | 3 | 1.07% | GO:0001071 |
|  | antioxidant activity | 2 | 0.71% | GO:0016209 |
| **Cellular component** | symplast | 3 | 1.07% | GO:0055044 |
|  | extracellular matrix | 1 | 0.36% | GO:0031012 |
|  | organelle | 57 | 20.36% | GO:0043226 |
|  | cell part | 91 | 32.50% | GO:0044464 |
|  | membrane-enclosed lumen | 2 | 0.71% | GO:0031974 |
|  | membrane part | 34 | 12.14% | GO:0044425 |
|  | extracellular region | 14 | 5.00% | GO:0005576 |
|  | cell junction | 3 | 1.07% | GO:0030054 |
|  | membrane | 47 | 16.79% | GO:0016020 |
|  | macromolecular complex | 32 | 11.43% | GO:0032991 |
|  | cell | 91 | 32.50% | GO:0005623 |
|  | organelle part | 18 | 6.43% | GO:0044422 |
| **Bological process** | reproduction | 3 | 1.07% | GO:0000003 |
|  | multi-organism process | 6 | 2.14% | GO:0051704 |
|  | developmental process | 13 | 4.64% | GO:0032502 |
|  | positive regulation of biological process | 5 | 1.79% | GO:0048518 |
|  | multicellular organismal process | 14 | 5.00% | GO:0032501 |
|  | cellular process | 151 | 53.93% | GO:0009987 |
|  | single-organism process | 134 | 47.86% | GO:0044699 |
|  | growth | 4 | 1.43% | GO:0040007 |
|  | signaling | 10 | 3.57% | GO:0023052 |
|  | negative regulation of biological process | 5 | 1.79% | GO:0048519 |
|  | metabolic process | 183 | 65.36% | GO:0008152 |
|  | biological regulation | 37 | 13.21% | GO:0065007 |
|  | reproductive process | 11 | 3.93% | GO:0022414 |
|  | immune system process | 1 | 0.36% | GO:0002376 |
|  | regulation of biological process | 35 | 12.50% | GO:0050789 |
|  | cellular component organization or biogenesis | 23 | 8.21% | GO:0071840 |
|  | rhythmic process | 2 | 0.71% | GO:0048511 |
|  | response to stimulus | 29 | 10.36% | GO:0050896 |
|  | localization | 36 | 12.86% | GO:0051179 |

**References**

Gonsalves, D. (2004). Transgenic Papaya in Hawaii and Beyond. *AgBioForum* 7**,** 36-40.

Ming, R., and Moore, P.H. (2014). *Genetics and Genomics of Papaya.* New York: Springer.
